# Differential directional effects between insomnia symptoms and suicidal ideation across trait depression levels: a cross-lagged network analysis among Chinese medical undergraduates

**DOI:** 10.3389/fpsyt.2025.1581827

**Published:** 2025-05-21

**Authors:** Jingxuan Zhang, Xiaolin Zhang, Feng Hu, Kuiliang Li, Mengjie Luo, Yang Yu, Zhengzhi Feng

**Affiliations:** ^1^ School of Psychology, Army Medical University, Chongqing, China; ^2^ Experimental Research Center of Medical and Psychological Science (ERC-MPS), School of Psychology, Army Medical University, Chongqing, China; ^3^ Teaching Examination Center, Army Medical University, Chongqing, China; ^4^ Department of Medical Psychology, The Southwest Hospital, Army Medical University, Chongqing, China; ^5^ Psychological Training Center, School of Psychology, Army Medical University, Chongqing, China; ^6^ School of Psychology, Shaanxi Normal University, Xi’an, China; ^7^ School of Basic Medicine, Army Medical University, Chongqing, China

**Keywords:** insomnia, suicidal ideation, trait depression, cross-lagged panel network, dynamic relationships

## Abstract

**Background:**

Suicidal ideation (SI) is intricately linked with insomnia and trait depression, yet the directional relationships and the role of trait depression remain unclear. This study sought to investigate the dynamic interplay between insomnia symptoms, SI, and trait depression (including trait anhedonia (TAN) and trait dysthymia (TDY)), aiming to clarify the role of trait depression in the relationship between insomnia and SI.

**Methods:**

A longitudinal design was employed to assess 566 undergraduate students (aged 18-25) recruited from a medical university in China. Participants underwent comprehensive assessments with a one-month interval between baseline and follow-up, applying the Athens Insomnia Scale (AIS), the Self-rating Idea of Suicide Scale (SIOSS), and the Trait Depression Scale (TDS). Cross-lagged panel network (CLPN) models were implemented to examine temporal associations, centrality metrics, and network differences between high/low TAN and TDY subgroups. Network stability was evaluated using bootstrap methods.

**Results:**

Insomnia symptoms, particularly AIS6 (sense of well-being during the day) and AIS7 (functioning), emerged as pivotal nodes significantly predicting SI factors, including despair (DSP) and suicide (SUI), with bidirectional feedback observed. TAN emerged as a central node, strongly influenced by insomnia and SI. TDY primarily influenced TAN and optimism (OPT). In the high-TAN group, OPT was a mediator among the nodes, OPT, AIS2 (awakening during the night), and AIS7 were key bridging nodes, whereas AIS3 (final awakening earlier than desired), AIS8 (sleepiness during the day), and DSP bridged in the low-TAN group. High/low TDY networks exhibited structural congruence but significant differences in bridge centrality rankings.

**Conclusion:**

Insomnia symptoms exacerbate SI by impairing daytime functioning and emotional regulation, with trait anhedonia serving as a critical node. Personalized interventions targeting specific insomnia symptoms (e.g., AIS6, AIS7 or AIS8) and suicidal emotional factors (e.g., OPT or DSP) are crucial disrupting feedback loops or critical connections to reduce suicide risk, particularly in individuals with varying levels of trait anhedonia. Although medical undergraduates represent a population commonly affected by mental health problems, the specialized nature of our sample may limit the generalizability of our findings. Future research and validation should be conducted in more diverse populations.

## Introduction

Suicide is a multifaceted public health issue that arises from the interplay of multiple psychological, physiological, and social factors. The detection of suicidal ideation (SI) is a critical strategy to evaluate suicide risk and to inform targeted prevention strategies for suicidal behaviors. Recent studies have increasingly highlighted the close associations between suicide risk, sleep disturbances, depressive symptoms, and specific personality traits (1–3). However, the directed relationships among them are not clear. Elucidating these dynamics is essential to enhancing the efficacy of suicide prevention efforts and developing targeted intervention strategies. This study aims to systematically investigate the complex interplay among these constructs, with a particular focus on the role of depressive traits in the relationship between insomnia and suicidal ideation, examining whether depressive traits function as a direct predictor of both insomnia and suicidal ideation or as a moderator of their association.

Sleep disorders, including insomnia, sleep fragmentation, and nightmares, have been consistently associated with suicide risk in multiple studies. For example, research among high-risk adolescents demonstrated that sleep problems, such as shorter total sleep time and longer sleep onset latency, indirectly contribute to suicidal thoughts through increased consummatory anhedonia (4). Additionally, a longitudinal study on COVID-19 patients revealed that poor sleep quality influences suicide risk via a cascade of mediating pathways involving anxiety and depressive symptoms (5). Collectively, these findings suggest that sleep disorders are not only an independent predictive factor for suicide risk but may also amplify suicide risk by impairing emotional and cognitive functions. Among various sleep problems, insomnia has been found to have the strongest association with suicidal ideation (6). Research indicates that insomnia, especially difficulties with sleep onset, frequent nighttime awakenings, or early morning awakenings, is significantly correlated with suicidal thoughts, plans, and attempts (6, 7). However, evidence shows positively connection between nightmare and SI, but mixed outcome between insomnia symptoms and suicide risk, meaning that relationship between insomnia severity and suicidal ideation level remain unclear (7). Nevertheless, compared to other sleep disorders, current evidence suggests that insomnia is most directly and closely linked with suicidal ideation (6). Focusing on insomnia, growing evidence has established it as a significant independent risk factor for suicidality, with recent studies highlighting its complex relationship with suicidal ideation and behaviors. A prospective cohort study demonstrated that insomnia, particularly in the context of short sleep duration, significantly elevates suicidality, both directly and indirectly through its impact on depression (8). Specifically, the findings showed that the total effect of insomnia on suicide risk was 2.85 times higher among short sleepers, with 32% of this effect mediated by depression. Furthermore, nightmares, often comorbid with insomnia, have been identified as an independent predictor of suicide, with persistent nightmares significantly increasing the risk of suicidal attempts. In young adults, both nightmares and depression have been demonstrated to mediate the relationship between insomnia and suicidal ideation, emphasizing the multifaceted nature of this association (9). These findings underscore the importance of addressing insomnia in suicide risk evaluation. Although meta-analysis has demonstrated that, among various sleep disturbances, insomnia and nightmares show the strongest associations with suicide risk (6), findings regarding the direction and consistency of the relationship between insomnia and suicide remain mixed and require further investigation (7). One possible explanation for these inconsistencies is that insomnia is not a unitary construct but is comprised of several distinct symptoms—such as difficulty initiating sleep, difficulty maintaining sleep, and early morning awakening—which may have differential associations with suicidal ideation and behaviors.

Existing evidence indicates that different insomnia symptoms usually exacerbate suicidality by impairing emotional regulation mechanisms. Specifically, depression plays an important role in the relation between insomnia and suicide. Research among patients with major depressive disorder (MDD) demonstrated that anhedonia plays a significant mediating role between sleep problems and suicidal thoughts. A separate investigation of the general population, depressive mood, high anger, abnormal sleep time, and family psychiatric history were identified as the main risk factors for suicidal ideation in the general population (10). These findings suggest that depressive mood is not only related to suicidal ideation but may also interact with other factors, like sleep problems, in increasing suicide risk. Further research revealed that depression functions as a mediator between insomnia and suicide. For instance, a study on adolescents demonstrated that poor sleep quality increases the risk of suicidal ideation through increased depressive symptoms (11). More precisely, short sleep time and poor sleep quality are significantly associated with suicidal ideation, suicide plans, and suicide attempts in male adolescents. This association attenuates after controlling for depressive symptoms (11). This indicates that depressive symptoms may play a mediating role between sleep disorders and suicide risk, that is, sleep disorders first lead to an increase in depressive symptoms, which in turn further increase suicide risk. Research among American veterans, childhood trauma indirectly influences sleep quality through increased depression, impulsivity, and hostility traits. These factors collectively increase the suicide risk of veterans (12). This further supports the view of depressive symptoms as a mediating variable.

However, extensive research has demonstrated that depression and insomnia have bidirected predictive relationships (2). Therefore, the role of depression in the relationship between insomnia and suicide warrants further investigation. Emerging evidence suggests that depressive symptoms not only act as mediating variables but also as traits that persistently modulate the relationship between sleep and suicide risk. For example, research conducted in the general population revealed that even after controlling for other variables, depressive mood remains significantly related to suicidal ideation, suggesting that depressive mood may act as a trait that consistently influences the relationship between sleep and suicide risk (10). However, in another study on patients with major depressive disorder, recent changes in anhedonia are significantly related to the higher risk of suicidal ideation, while lifelong anhedonia is not (13). This highlights that whether depression as a trait exerts a significant influence between sleep disorders and suicide risk remains inconsistent. This inconsistency may partly stem from the fact that anhedonia can influence suicide through multiple pathways, that not only affects emotions and cognitive functions but may also further increase suicide risk by changing the individual’s reward system function. Therefore, it is important to further investigate how trait-level depression acts as a stable predisposition influencing risk pathways, beyond what is indicated by general measures of depression.

The multifaceted nature of insomnia, depressive traits, and suicidal ideation likely contributes to the inconsistent results of their interrelationships. Therefore, studying insomnia at the symptom level, rather than as a single entity, could provide a more nuanced and accurate understanding of its relationship with suicide. To address this gap, our study employs network analysis at the symptom level to examine the precise links between specific insomnia symptoms and suicidal ideation. Additionally, addressing the key symptom-level intervention targets needs causal analysis. Most prior studies have relied on static associations or simple mediation models, which fail to capture the complex temporal interplay between variables. Although some longitudinal studies (4, 11, 14–16) have sought to elucidate the causal relations between them, the identification of specific symptoms as key intervention targets remains elusive. This level of analyses may guide personalized interventions targeting high-risk individuals. Therefore, we integrate longitudinal design and network analysis to address this gap.

Cross-lagged panel network (CLPN) model (17) is an advanced statistical approach that integrates the principles of cross-lagged panel models with network analysis to examine the dynamic, directional relationships among multiple variables—typically symptoms—across different time points (18, 19). Unlike traditional cross-lagged panel models, which focus on the relationships between latent variables or composite scores, CLPN allows researchers to investigate the temporal interplay between individual symptoms within a network framework. This approach provides a more granular understanding of how specific symptoms influence each other over time, capturing both autoregressive effects (the stability of symptoms) and cross-lagged effects (the influence of one symptom on another at a subsequent time point). In this study, we used CLPN to model the interrelationships among the symptoms of insomnia, depressive traits, and suicidal ideation, trying to reveal the directional effects within the network, identifying key symptoms to enhance the precision of targeted interventions. Specifically, we focus on key network metrics such as in-expected influence (in-EI), out-expected influence (out-EI), and bridge expected influence (bridge-EI), which quantify the centrality and bridging roles of symptoms in the temporal network. Of particular importance are depressive traits, as it is essential to determine whether they function as predictors or moderators. To address this, we construct two distinct network models to compare and elucidate the precise role of depressive traits.

## Materials and methods

### Participants

#### Inclusion and exclusion criteria

The inclusion criteria were: (a) currently enrolled medical undergraduates; (b) age 18 or older; (c) provision of informed consent. Exclusion criteria included: (a) self-reported current diagnosis of any severe psychiatric disorder; (b) significant medical conditions that could confound psychological assessment; (c) being on a leave of absence during the survey period.

#### Initial recruitment

A total of 674 undergraduate students aged 18 to 25 years old were randomly recruited from a medical university in Chongqing. Participants underwent assessments of insomnia, trait depression, and suicidal ideation at the first wave (Wave 1). Baseline demographic and background data were collected exclusively at Wave 1, including gender, age, only-child status, family structure, parenting style, interpersonal relationships, and left-behind child.

#### Following-up

A month later, follow-up assessments were conducted one month later (Wave 2). Of the initial cohort, 617 participants completed Wave 2 assessments, among whom 51 were excluded due to elevated scores on the lying subscale of the suicidal ideation measure. Consequently, 566 participants aged 18 to 25 (median =19.00, interquartile range =1.00) years remained for further analyses, including 443 males.

#### Ethical statement

This study was approved by the Medical Ethics Committee of Army Medical University, and all procedures conformed to the Declaration of Helsinki. Written informed consent was obtained from all participants prior to participation.

### Measurements

#### Insomnia

The Athens Insomnia Scale (AIS) was utilized to assess the severity of insomnia symptoms among participants. Developed by Soldatos et al. (20), the AIS is a self-reported questionnaire developed to assess insomnia severity based on the International Classification of Diseases, 10th Revision (ICD-10) criteria. The scale consists of 8 items, with the first 5 items assessing nocturnal sleep disturbances and the remaining 3 items assessing daytime dysfunction. Each item is scored on a 4-point Likert scale ranging from 0 (“no problem at all”) to 3 (“very severe problem”), yielding a total score ranging from 0 to 24. Higher scores indicate more severe insomnia symptoms. The AIS exhibited strong reliability with Cronbach’s α of 0.853 (Wave 1) and 0.915 (Wave 2) in this study.

#### Suicidal ideation

The Self-rating Idea of Suicide Scale (SIOSS) was developed by Xia and Wang (21), consisting of 26 items, where higher scores reflect more severe suicidal ideation. It includes 4 factors including despair (DSP, 8 items), optimism (OPT, 5 items), sleep (SLP, 4 items), and suicide (SUI, 4 items), and a deception measure for validity (22). The scale demonstrated good reliability and validity for Chinese populations (21–23). In this study, following the exclusion of participants with elevated deception scores, it also had acceptable reliability with Cronbach’s α of 0.858 (Wave 1) and 0.889 (Wave 2).

#### Trait depression

The Trait Depression Scale (TDS) of the State-Trait Depression Scales (STDS) was utilized to assess trait depression. The STDS is a well-validated instrument designed to differentiate between state depression (transient emotional states influenced by situational factors) and trait depression (stable, enduring predispositions toward depressive affect) (24). The scale consists of 2 subscales: the State Depression Scale (SDS) and the Trait Depression Scale (TDS), each containing 16 items in the Chinese version (25). Responses are rated on a 4-point scale, ranging from 1 (not at all) to 4 (very much so), with higher scores indicating greater levels of depressive symptoms. The TDS comprises 2 factors: trait anhedonia (TAN, 8 items) and trait dysthymia (TDY, 8 items). In the present study, the TDS demonstrated strong reliability, with Cronbach’s α of 0.951 (Wave 1) and 0.961 (Wave 2).

### Statistical analyses

#### Descriptive statistics

Descriptive statistics and demographic characteristics were analyzed using IBM SPSS 25.0 (26).

#### Network analyses

To examine the temporal dynamics and directional relationships among variables, the cross-lagged panel network (CLPN) analysis (17) was performed using R 4.4.1 (27) in RStudio 2024.04.2 (28). The analysis was performed in 2 parts: CLPN analyses with the 2 factors of trait depression as nodes and as grouping variables to stratify individuals into low and high levels of trait anhedonia or trait dysthymia. Data were collected at two time points (Wave 1 and Wave 2), and covariates were included to control for potential confounders. Among the network nodes, covariates were controlled and excluded from network visualization. Regularized cross-lagged effects were estimated via the least absolute shrinkage and selection operator (LASSO) regression (29) via the “glmnet” package (30). The regularization parameter (λ) was determined through 10-fold cross-validation to optimize model fit and sparsity. The resulting adjacency matrices were visualized using the “qgraph” package (31). Nodes were grouped into meaningful clusters (e.g., “Insomnia” and “Suicidal Ideation”), and edge weights represented the strength and direction of cross-lagged effects. Node centrality metrics, including in expected influence (in-EI, how strongly other variables predict a given node), out expected influence (out-EI, how strongly a given node predicts other variables), and bridge expected influence (bridge-EI, how strongly a given node links between two constructs), were calculated to determine the most influential variables in the network (32). The stability of the network structure and centrality indices was assessed through non-parametric bootstrapping with 1,000 iterations via the “bootnet” package (18). Case-dropping bootstrap procedures were additionally performed to assess the robustness of the results, with a recommended correlation stability (CS) coefficient threshold of ≥0.25, ideally >0.5 (18). Bridge expected influence was calculated to identify nodes that connected distinct clusters within the network (33). Network comparison tests were performed following the methodology outlined by Funkhouser et al. (19), to statistically compare the network structures between the low and high levels of trait anhedonia and trait dysthymia.

## Results

### Demographic characteristics and descriptive statistics

Descriptive statistics for demographic characteristics and psychological measures are presented in **Table 1**. Since the variable “age” and all psychological measures deviated from normality based on the Kolmogorov-Smirnov test (D: 0.120 to 0.511, *p <*0.05), median and interquartile range (IQR) were used to characterize their distribution.

**Table 1:** Descriptive statistics of the demographic information and the psychological measures.

Variables (code)	N (1076)	%	Median (IQR)
Gender			
female (1)	123	21.73	
male (2)	443	78.27	
Age			19.00 (1.00)
Only-child			
yes (1)	314	55.5	
no (2)	252	44.5	
Family structure			
two parents (1)	523	92.4	
one parent (2)	32	5.7	
multi-generation (3)	3	0.5	
others (4)	8	1.4	
Parenting style			
authoritarian (1)	44	7.8	
permissive (2)	78	13.8	
indulgent (3)	6	1.1	
authoritative (4)	438	77.4	
Interpersonal relationships			
more than 3 good friends (1)	459	81.1	
1 or 2 good friends (2)	102	18.0	
no good friend (3)	5	0.9	
Left-behind child			
yes (1)	122	21.6	
no (2)	444	78.4	
Psychological measures of Wave 1			
TDS			24.00 (14.00)
*TAN*			14.00 (9.00)
*TDY*			10.00 (7.00)
AIS			4.00 (6.00)
SIOSS			2.00 (4.00)
*DSP*			1.00 (3.00)
*OPT*			0.00 (0.00)
*SLP*			0.00 (1.00)
*SUI*			0.00 (0.00)
Psychological measures of Wave 2			
TDS			24.00 (16.00)
*TAN*			15.00 (9.00)
*TDY*			10.00 (7.00)
AIS			5.00 (6.00)
SIOSS			2.00 (5.00)
*DSP*			1.00 (3.00)
*OPT*			0.00 (0.00)
*SLP*			1.00 (2.00)
*SUI*			0.00 (0.00)

N, number of valid samples; IQR, interquartile range; Wave 1, timepoint 1; Wave 2, timepoint 2; TDS, the Trait Depression Scale; TAN, the trait anhedonia factor of TDS; TDY, the trait dysthymia factor of TDS; AIS, the Athens Insomnia Scale; SIOSS, the Self-rating Idea of Suicide Scale; DSP, the despair factor of SIOSS; OPT, the optimism factor of SIOSS; SLP, the sleep factor of SIOSS; SUI, the suicide factor of SIOSS.

### Cross-lagged panel network model

#### Network structure and cross-lagged edges

The resulting network revealed a complex pattern of temporal associations among the variables, with a modular organization. The network was partitioned into two distinct modules: AIS1-AIS8 comprising the “Insomnia” module, while DSP, OPT, SUI, and SLP comprising the “Suicidal Ideation” module. Notably, TAN and TDY, both dimensions of trait depression, were not co-clustered within the network. TAN occupied a central position in the network, highlighting its pivotal role. Meanwhile, AIS6 and SLP were also identified as critical variables as they were relatively close to the center of the network and exhibited dense in- and out-edges. While the overall network density was modest, a subset of variables displayed strongly interconnections, including AIS6, AIS7, DSP, SUI, SLP, TAN, and TDY, indicating robust mutual influences.

**Figure 1:**
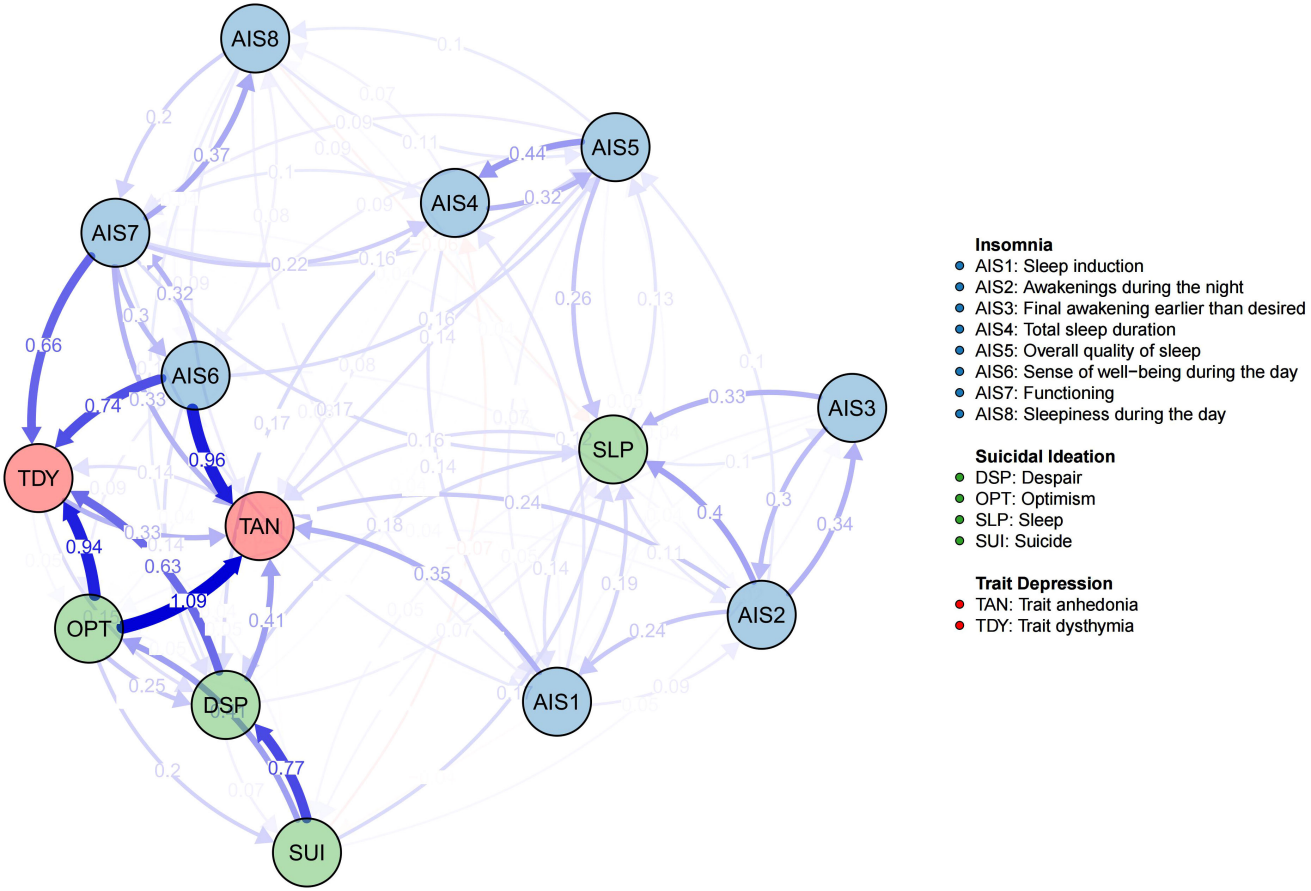
The network structure and cross-lagged effects. The blue edges represent positive weighted correlations, whereas the red edges represent negative weighted correlations. The darker the edge color is, the higher the edge weight is.

The cross-lagged relationships revealed several key dynamic patterns. AIS1 (β =0.348), AIS6 (β =0.961), AIS7 (β =0.331), DSP (β =0.413), OPT (β =1.094), and TDY (β =0.330) exhibited strong predictive influences on TAN, with relative high edge weights. Other notable directed relationships included AIS2→AIS3 (β =0.338), AIS5→AIS4 (β =0.443), AIS4→AIS5 (β =0.324), AIS7→AIS6 (β =0.302), AIS6→AIS7 (β =0.324), AIS7→AIS8 (β =0.373), SUI→DSP (β =0.770), SUI→OPT (β =0.412), AIS2→SLP (β =0.398), AIS3→SLP (β =0.329), AIS6→TDY (β =0.745), AIS7→TDY (β =0.657), DSP→TDY (β =0.630), and OPT→TDY (β =0.945). All “β values” represent directional edge weight coefficients from the starting nodes at Wave 1 to the ending nodes at Wave 2. Additionally, reciprocal effects were observed between AIS4 and AIS5, as well as AIS6 and AIS7, suggesting bidirectional relationships within specific network segments. These results underscore the directional and asymmetrical characteristics of the temporal associations within the network. The network structure and cross-lagged effects are visualized in **Figure 1**, with detailed edges weights provided in **Supplementary Table S1**.

#### Centrality

**Figure 2:**
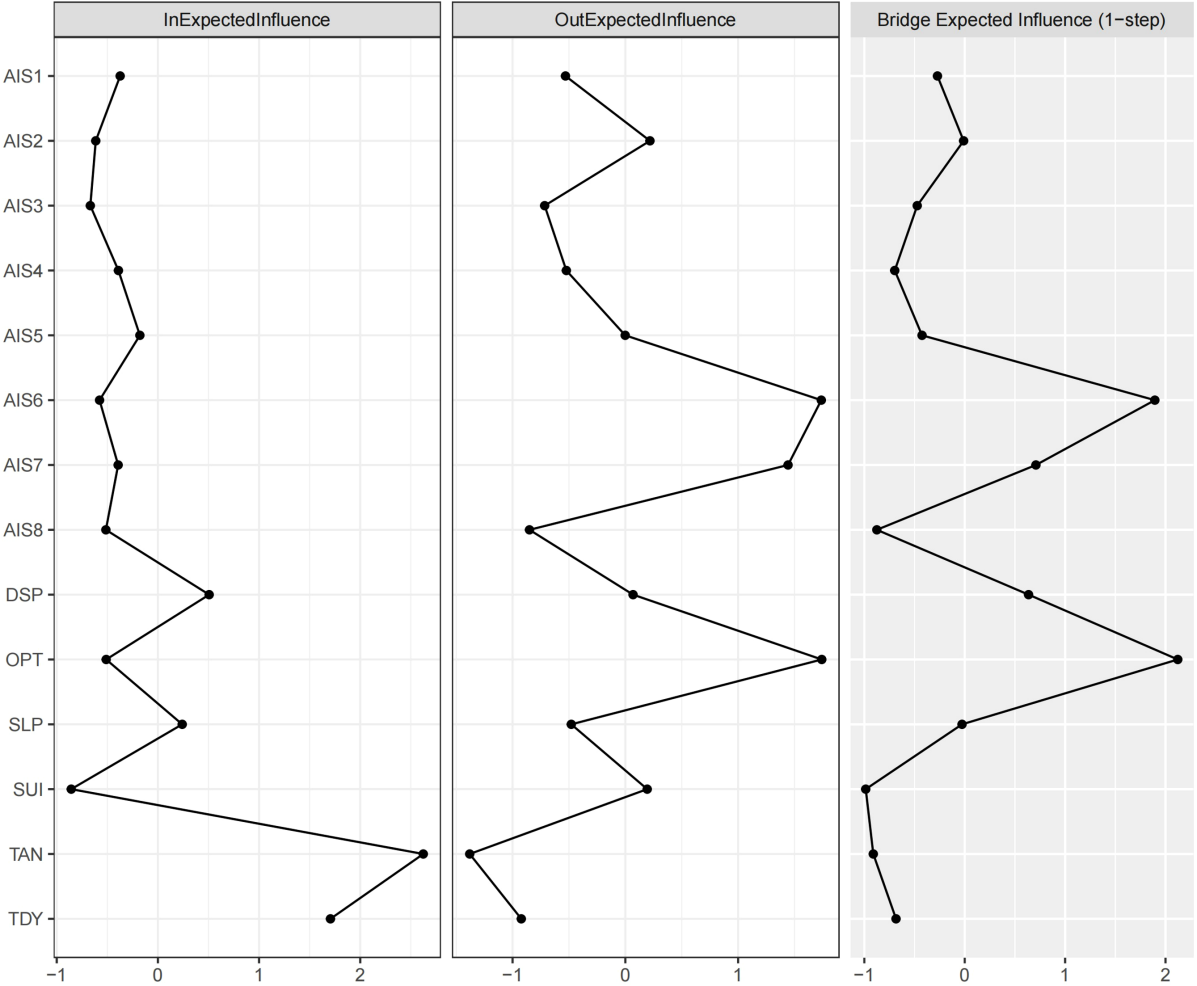
The centrality plot of the in-EI, out-EI, and bridge-EI for the network nodes. Refer to **Figure 1** for the meanings of the node labels. In this figure, x-axis represented the z-scores. The raw scores of the 3 EI indices were listed in **Supplementary Table S2**.

In the CLPN, nodes centrality typically comprises both in-EI and out-EI, which quantify the cumulative influence of edges directed to and from a node, respectively. Thus, out-EI reflects a node’s influence on others, while in-EI captures its susceptibility to influence from others. In this network, TAN and TDY exhibited relatively high in-EI values, indicating their strong predicted effects from other nodes. In contrast, AIS6, AIS7, and OPT emerged as key predictor variables with relatively high out-EI values. The above centrality results (see **Figure 2**) were consistent with the network structure visualized in **Figure 1**.

The nodes AIS6 and OPT exhibited relatively high bridge-EI values, highlighting their role in bridging distinct network communities (trait depression, insomnia, and suicidal ideation). Specifically, as illustrated in **Figure 1**, OPT was strongly influenced by SUI, while exerting a strong influence on TAN and TDY. This underscores OPT’s mediating role, confirming its function as a bridge node. A similar role was observed for AIS6, as it mediated the influence from AIS7 to TAN and TDY. Thus, OPT primarily bridged trait suicidal ideation and trait depression, whereas AIS6 connected insomnia and trait depression.

### Network comparisons of the high and low levels of trait depression

#### Grouping

The 566 participants were classified into two distinct clusters based on their average scores on TAN across the 2 time points, using K-medoids clustering. This yielded a high-TAN group consisting of 311 individuals and a low-TAN group consisting of 255 individuals. To evaluate the statistical significance of intergroup differences, a Wilcoxon rank-sum test was performed. A significant difference was observed between the high-TAN and low-TAN groups (W =79305, *p <*0.001). Then, we used the same procedure to divide the participants into 2 groups by high and low levels of TDY, among which the high-TDY and low-TDY groups consisted of 221 and 345 individuals, respectively, with significant differences in TDY scores (W =76245, *p <*0.001). These results confirm that the clustering effectively partitioned participants into meaningful subgroups characterized by distinct levels of trait anhedonia and trait dysthymia severity.

#### Networks of different trait anhedonia levels

The comparative analysis of the networks uncovered several key insights into their structural and statistical similarities. The edge weight correlation (ρ =0.541, *p <*0.001) revealed a moderate to strong positive correlation between the edge weights of the two networks, suggesting a significant overlap in connection strengths. This is further supported by the Jaccard similarity (J =0.993), which demonstrates a high degree of edge overlap between the networks. Notably, 99.3% of edges were replicated in both the high and low networks, highlighting a remarkable consistency in the structure of the two networks.

In terms of centrality measures, the out-EI correlation (ρ =0.909, *p <*0.001) revealed a very strong positive relationship, highlight the high consistency of outgoing centrality across the networks. However, the in-EI correlation (ρ =0.552, *p* =0.067) was weaker and lacked statistical significance, suggesting potential differences in incoming centrality. This discrepancy was further evident in the rank consistency metrics, where both out-EI and in-EI rank consistency values are low (25%), suggesting divergent centrality rankings between the networks. For bridge-EI, the correlation (ρ =0.72, *p* =0.011) was strong and statistically significant, indicating relative consistency in bridging centrality across the networks. However, the rank consistency for bridge-EI was low (16.7%), implying variability in the relative importance of bridge nodes between the networks.

**Figure 3:**
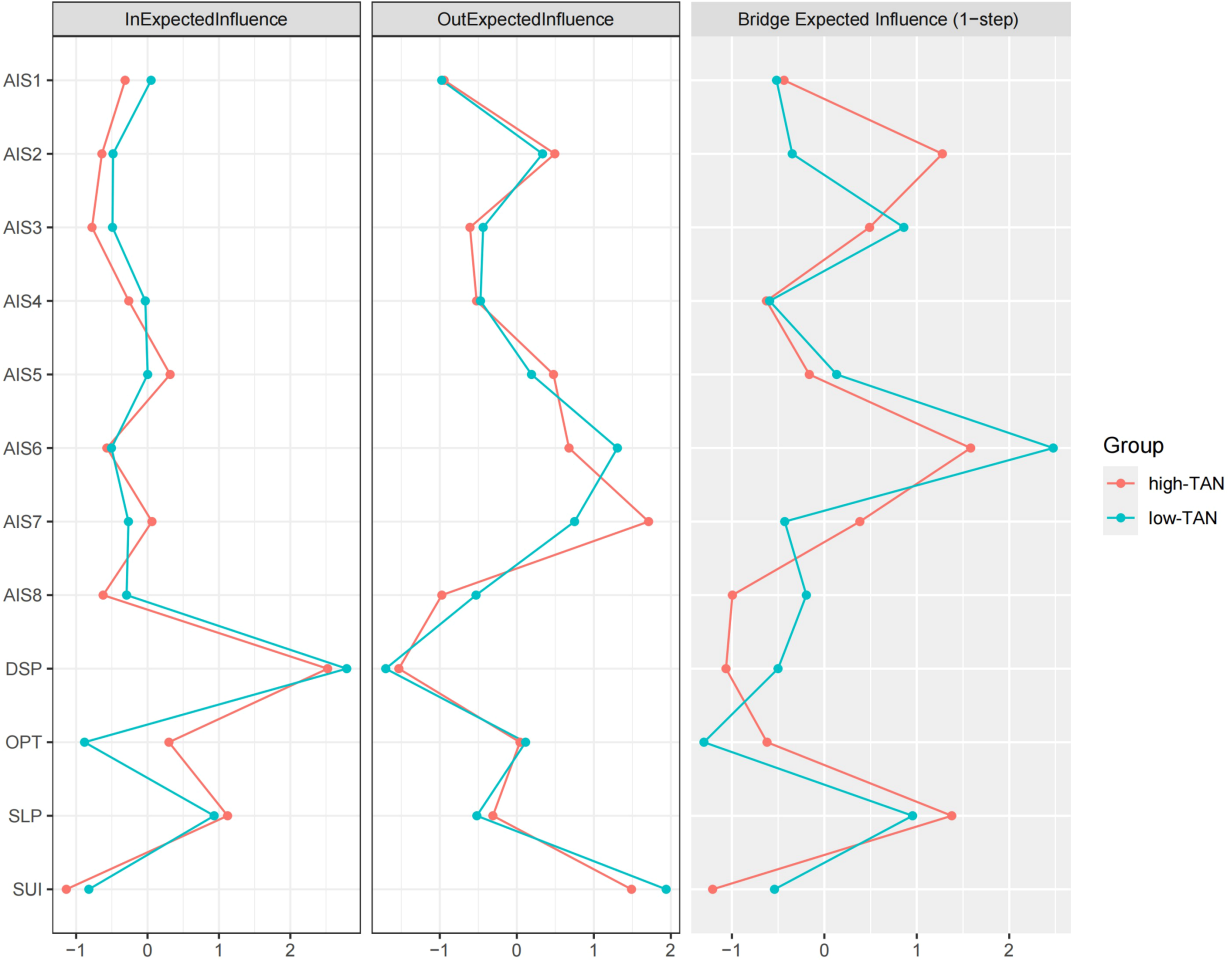
The EI values of high-TAN and low-TAN groups. Refer to **Figure 1** for the meanings of the node labels. In this figure, x-axis represented the z-scores. The raw scores of the EI indices were listed in **Supplementary Table S5**.

**Figure 4:**
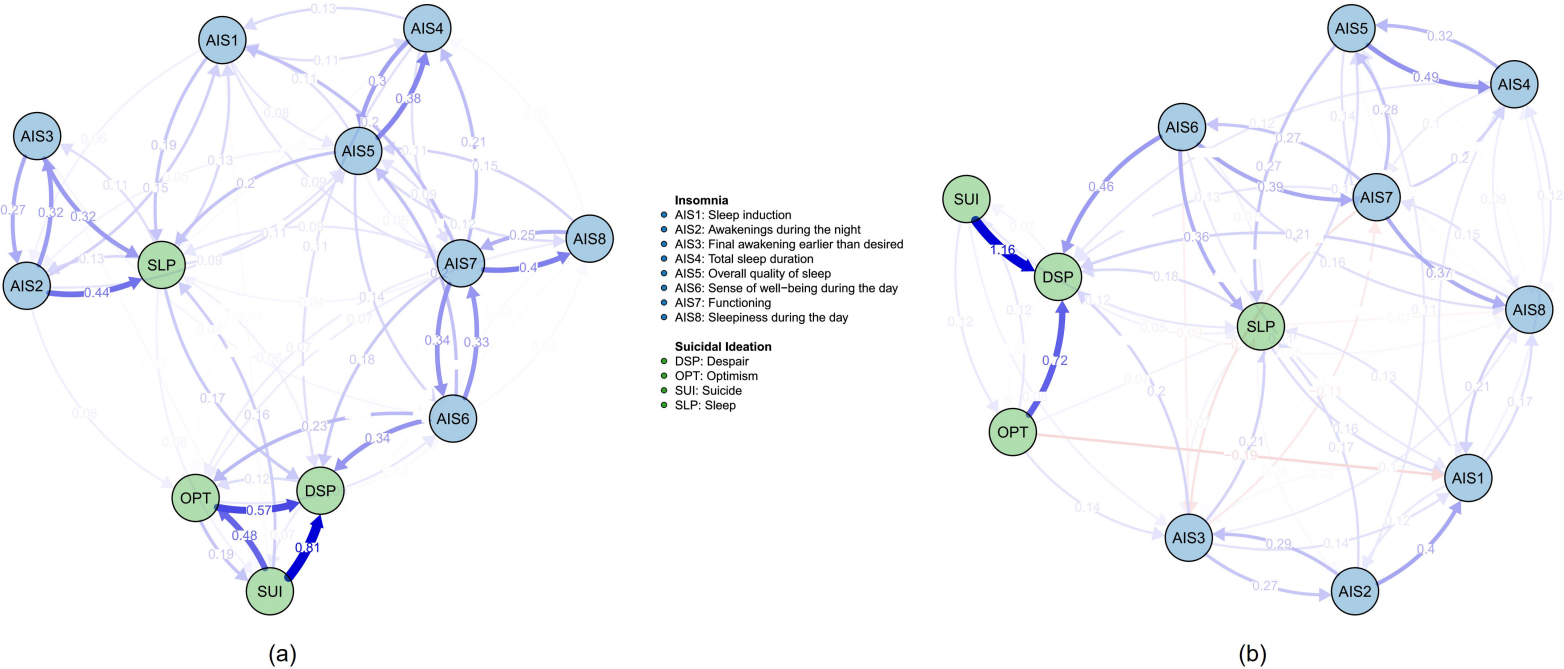
The network structure and cross-lagged effects of high-TAN and low-TAN groups. **(a)** high-TAN network; **(b)** low-TAN network. All the edges weights were listed in the **Supplementary Tables S3** and **S4**. Other notes refer to **Figure 1**.

Overall, these results suggest that the two networks exhibit a high degree of structural similarity in edge weights and edge replication. However, notable differences were observed in centrality and bridging properties. Specifically, as shown in **Figure 3**, the in-EI of OPT might have contributed to the non-significant correlation. According to **Figure 4**, this may be attributed to differences in the directional edge from SUI to OPT between the high and low trait anhedonia groups. Regarding centrality rankings, bridge-EI exhibited significant differences across the following nodes: AIS2, AIS3, AIS7, AIS8, DSP, and OPT. This suggests that AIS2, AIS7, and OPT were more influential in the high trait anhedonia group, whereas AIS3, AIS8, and DSP played a more prominent role in the low-level group.

#### Networks of different trait dysthymia levels

The comparative analysis of the high trait dysthymia and low trait dysthymia networks reveals critical insights into their structural and statistical similarities and differences. The edge weight correlation (ρ =0.572, *p <*0.001) demonstrates a robust positive correlation, highlighting the high consistency of connection strengths between nodes across both networks. This strong correlation is further supported by the Jaccard similarity (J =0.993), indicating nearly complete overlap between the two networks. Remarkably, 99.3% of edges were replicated in both the high and low dysthymia networks, underscoring their remarkable structural congruence. This implies that the core network architecture remains largely consistent irrespective of dysthymia levels.

In the analysis of centrality measures, the out-EI correlation (ρ =0.916, *p <*0.001) was exceptionally strong, signifying that the outgoing centrality of nodes is nearly identical in both networks. This implies that nodes’ influence in terms of outgoing connections is highly preserved across the high and low dysthymia conditions. In contrast, the in-EI correlation (ρ =0.72, *p* =0.011), though significant, was relatively weaker, suggesting variability in incoming centrality. This difference was further evident in the rank consistency metrics, with the out-EI rank consistency (41.7%) significantly higher than the in-EI rank consistency (33.3%). This indicates that while rankings based on outgoing centrality are stable, rankings based on incoming centrality exhibit greater variability. The analysis of bridge-EI revealed a strong and statistically significant correlation (ρ =0.797, *p* =0.003), highlighting the consistency of bridging centrality across the two networks. However, bridge-EI rank consistency was notably low (8.3%), which implies that while the overall bridging properties of nodes are similar, the relative importance of nodes as bridges varies significantly between the high and low dysthymia networks. This discrepancy implies that the role of specific nodes in mediating connectivity between network modules may differ with dysthymia levels.

**Figure 5:**
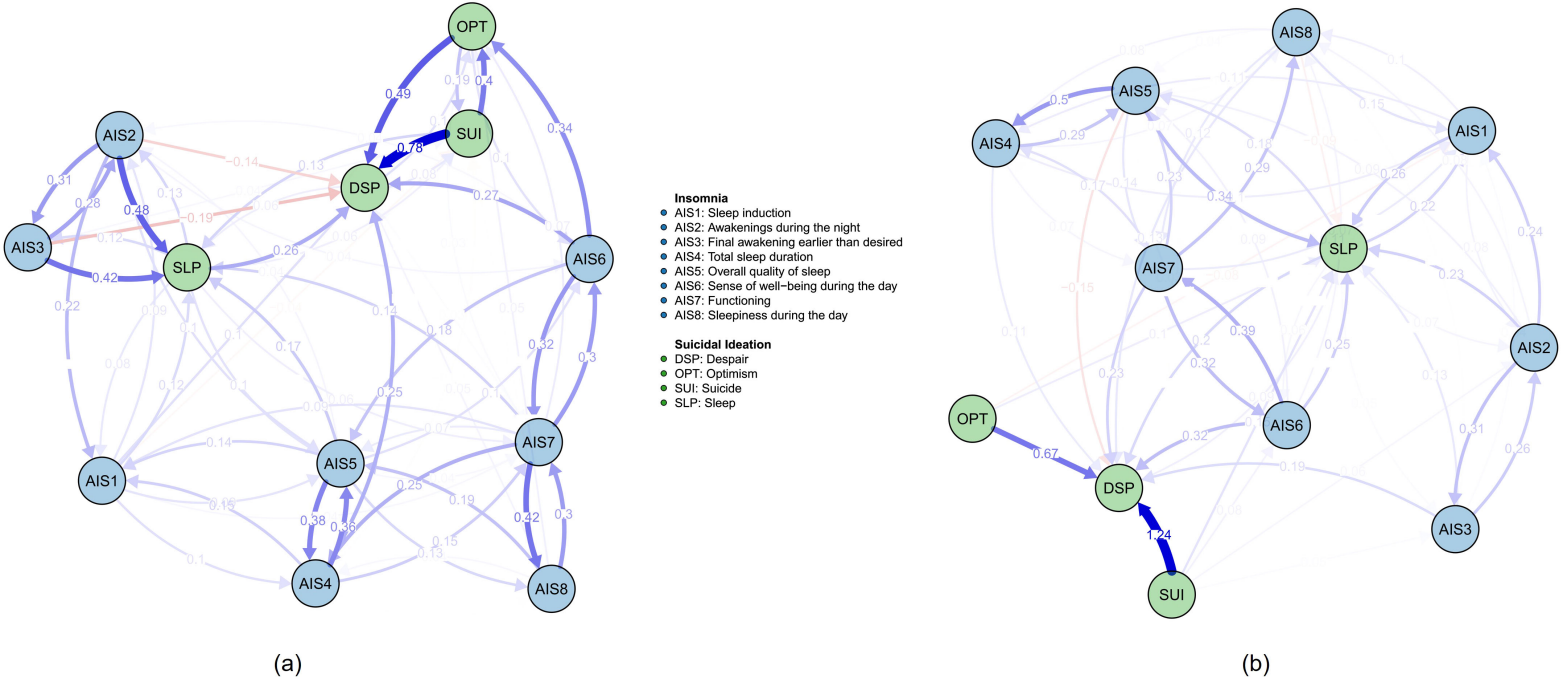
The network structure and cross-lagged effects of high-TDY and low-TDY groups. **(a)** high-TDY network; **(b)** low-TDY network. All the edges weights were listed in the **Supplementary Tables S6** and **S7**. Other notes refer to **Figure 1**.

**Figure 6:**
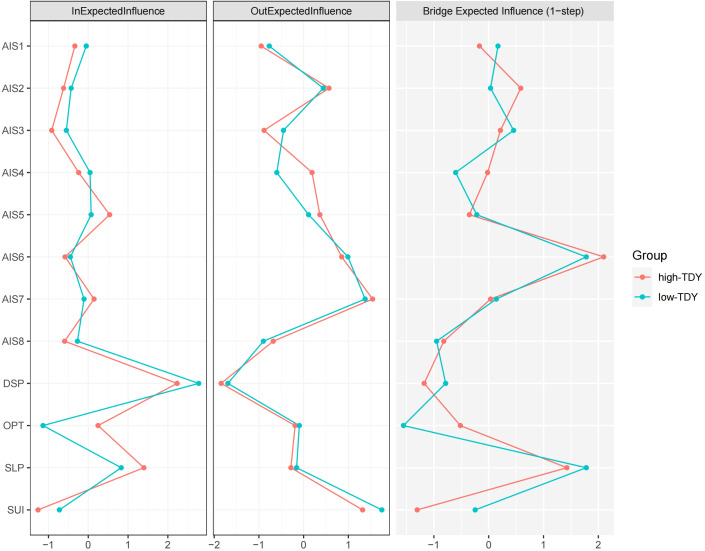
The EI values of high-TDY and low-TDY groups. Refer to **Figure 1** for the meanings of the node labels. In this figure, x-axis represented the z-scores. The raw scores of the EI indices were listed in **Supplementary Table S8**.

The high and low dysthymia networks exhibit a remarkable degree of structural similarity, especially in edge weights and edge replication. However, there are notable differences in centrality measures, especially in incoming centrality and bridging properties. These findings suggest that while the core structure of the networks remains consistent, the functional roles of certain nodes, particularly in incoming influence and bridging, may vary with dysthymia levels. Particularly, as illustrated in **Figure 5**, the incoming edge from SUI to OPT differed between the two networks, consistent with the in-EI plot in **Figure 6**. However, the high in-EI correlation between the two groups suggested that this variance did not substantially alter the overall network structure. Although in-EI and out-EI rank consistency remained low, they were relatively high compared to the consistency between high and low trait anhedonia groups, suggesting that trait anhedonia may exert a stronger influence on nodes’ relative importance than trait dysthymia. Nevertheless, bridge-EI rank consistency results indicated that trait dysthymia had a stronger influence on nodes’ connectivity between network modules than trait anhedonia.

Additionally, we have found a consistent pattern in our subgroup analyses (such as comparisons between high-TAN and low-TAN or high-TDY and low-TDY groups). AIS6 (“sense of well-being during the day) directly influenced DSP, while AIS7 exerted its effect on DSP via AIS6; DSP served as the core of suicidal ideation and was also significantly influenced by the SUI factor. The directional paths were listed in **Table 2**.

**Table 2:** The directional paths among AIS6, AIS7, DSP, and SUI.

Groups	Path and coefficients
high-TAN	AIS7 → ^0.338^ → AIS6 → ^0.339^ → DSP ← ^0.807^ ← SUI
low-TAN	AIS7 → ^0.273^ → AIS6 → ^0.459^ → DSP ← ^1.156^ ← SUI
high-TDY	AIS7 → ^0.305^ → AIS6 → ^0.272^ → DSP ← ^0.779^ ← SUI
low-TDY	AIS7 → ^0.321^ → AIS6 → ^0.323^ → DSP ← ^1.244^ ← SUI

TAN, the trait anhedonia factor of the Trait Depression Scale; TDY, the trait dysthymia factor of the Trait Depression Scale; AIS6, an item of the Athens Insomnia Scale, meaning "sense of well-being during the day"; AIS7, an item of the Athens Insomnia Scale, meaning "functioning"; DSP, the despair factor of the Self-rating Idea of Suicide Scale; SUI, the suicide factor of the Self-rating Idea of Suicide Scale.

### Network stability and accuracy

#### Accuracy of the edges

**Figure 7:**
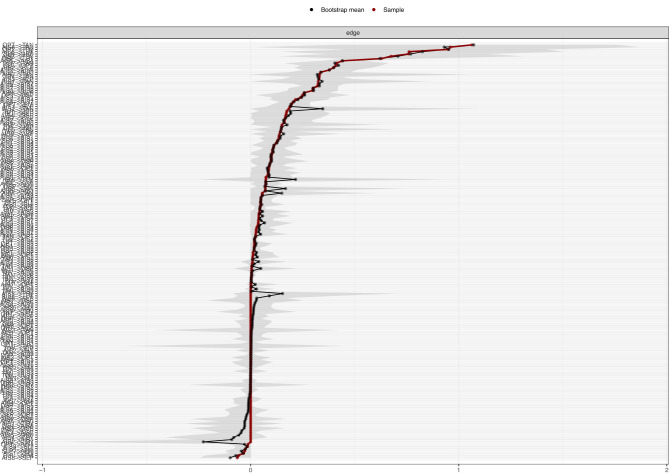
Bootstrap means of the edge weights for non-grouped network.

**Figure 8:**
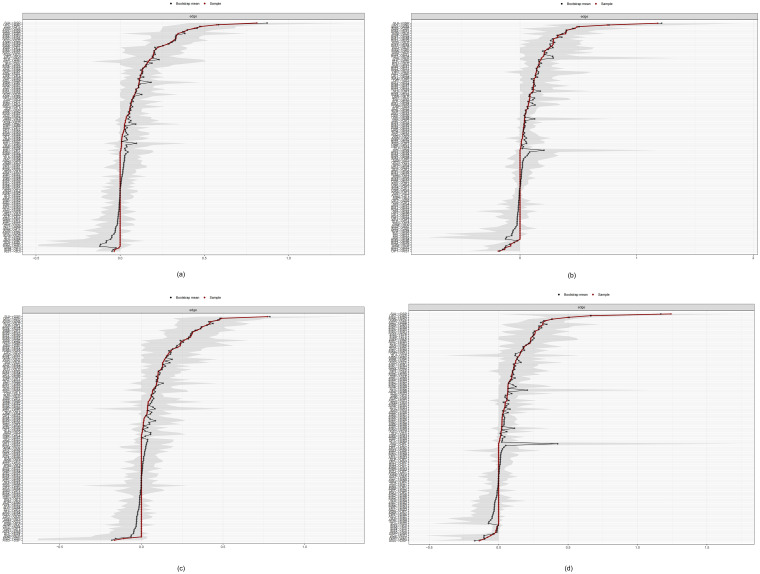
Bootstrap means of the edge weights for the grouped network. **(a)** high-TAN group; **(b)** low-TAN group; **(c)** high-TDY group; **(d)** low-TDY group.

Using bootstrapping method with 1,000 iterations, a nonparametric analysis of network edge accuracy was performed. The results revealed robust and consistent performance for the non-grouped network (**Figure 7**) and across different groups (**Figure 8**) as evidenced by the close alignment of the red line with the black dots, indicating a close correlation between the bootstrap means and the original sample edge weights. However, in the low trait dysthymia group, one edge (SUI→OPT, see **Supplementary Figure S2**) exhibited highly unstable weights across bootstrap samples. Additionally, several edges from SUI to other nodes (e.g. SUI→AIS6, SUI→AIS8, and SUI→SLP in low trait anhedonia group; SUI→AIS6 and SUI→SLP in low dysthymia group) showed wide 95% confidence intervals (CIs) (see **Supplementary Figures S1** and **S2**), suggesting that the effects of suicidal ideation on insomnia may be less pronounced in participants with low trait depression compared to those with high trait depression.

#### Stability of the centralities

**Figure 9:**
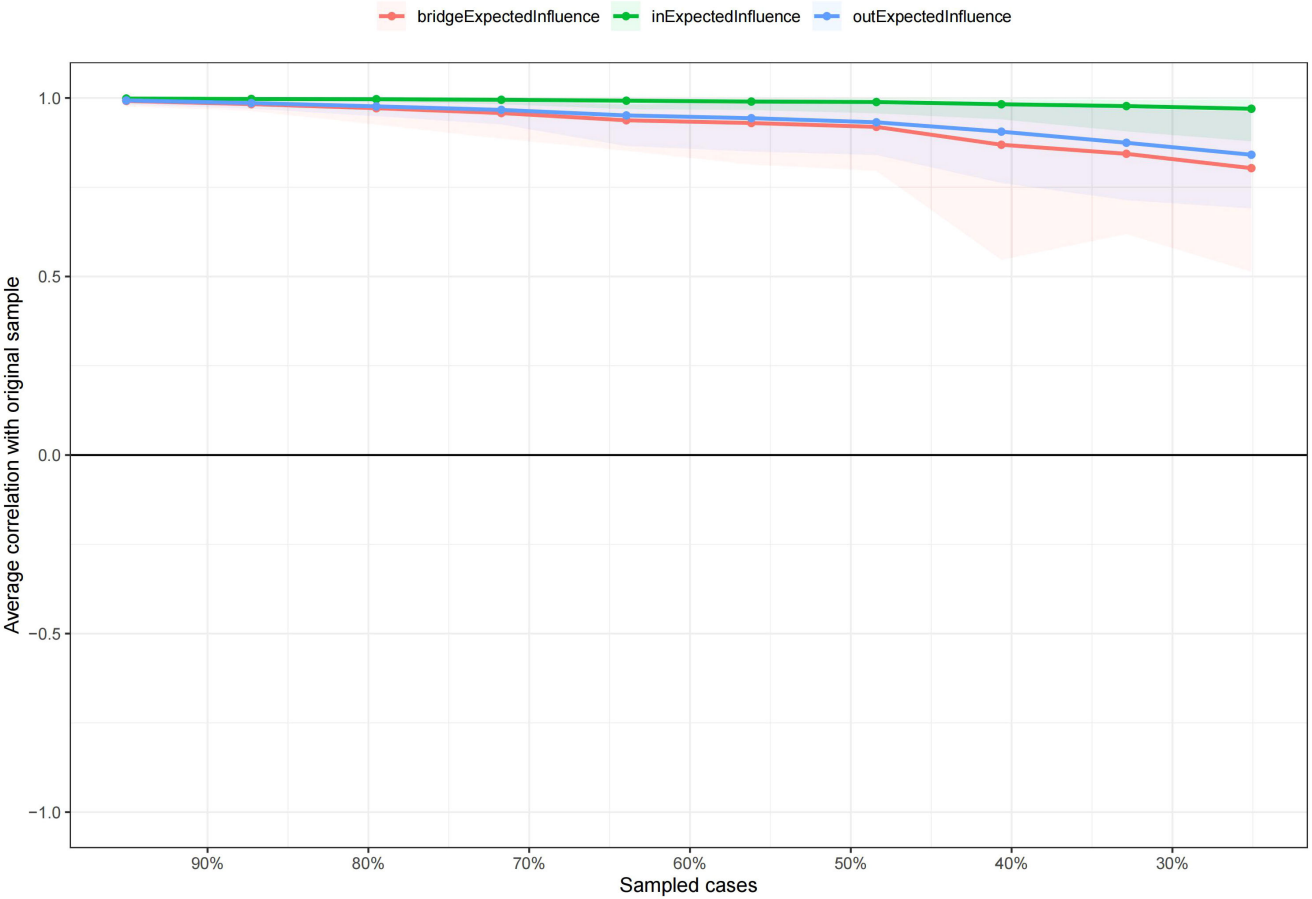
Case-dropping bootstrap results for non-grouped network.

**Figure 10:**
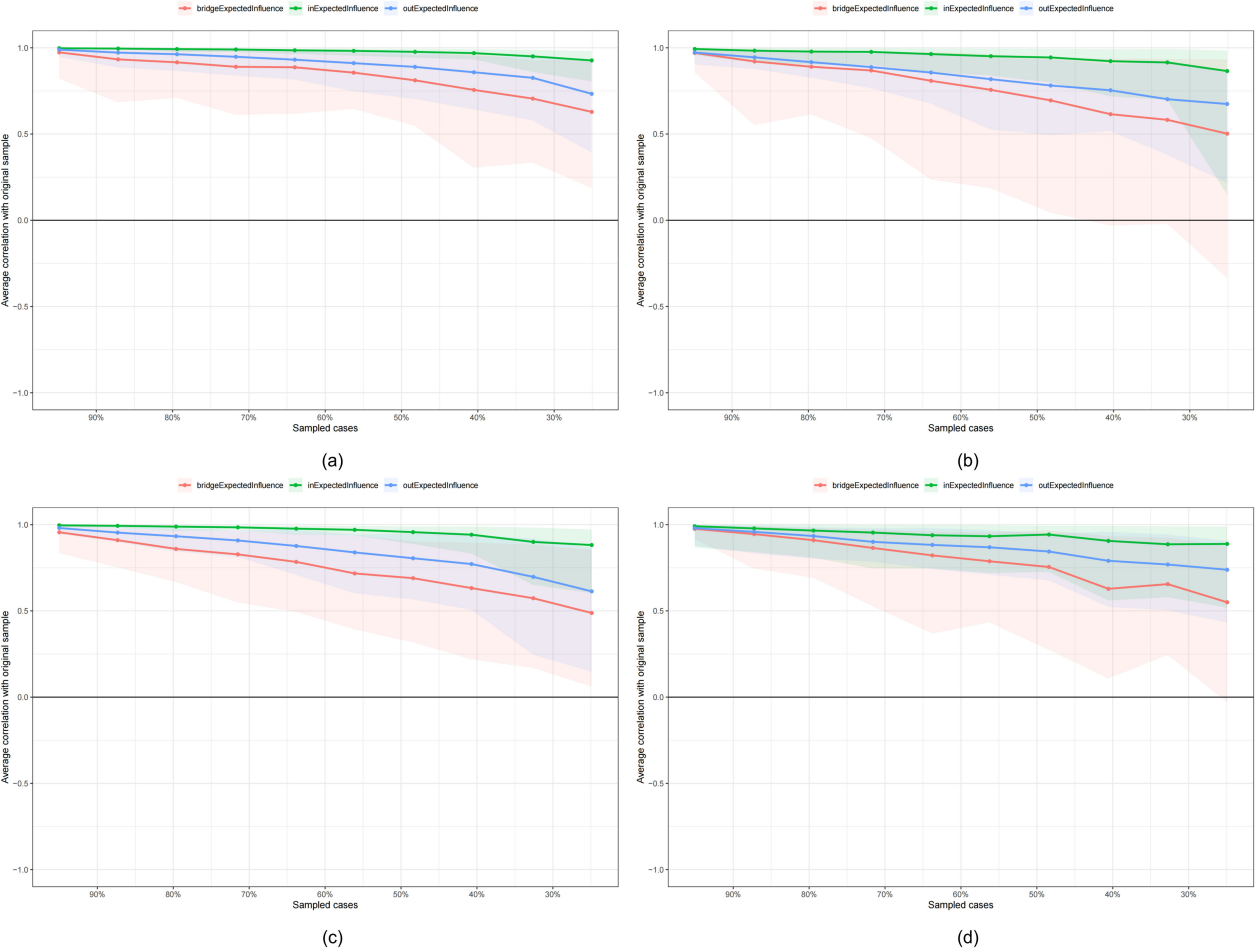
Case-dropping bootstrap results for grouped network. **(a)** high-TAN group; **(b)** low-TAN group; **(c)** high-TDY group; **(d)** low-TDY group.

The stability of the centralities, as assessed by the correlation stability (CS) coefficient resulted from case-dropping bootstrap, was evaluated for both grouped and non-grouped CLPN. For the grouped CLPN, comprising high-TAN and low-TAN or high-TDY and low-TDY subgroups, the in-EI values demonstrated robust stability, with CS coefficients ranging from 0.671 to 0.751, all exceeding the recommended threshold of 0.25. Similarly, the out-EI values exhibited acceptable stability, with coefficients ranging from 0.361 to 0.595. However, the bridge-EI values of all the subgroups were unstable, with coefficients consistently below 0.25 across all subgroups. This suggests that the variances of bridge-EI could not be reliably interpreted. In the non-grouped CLPN, both in-EI and out-EI values yielded CS coefficients of 0.749, indicating high stability. The bridge-EI, with a coefficient of 0.516, also surpassed the recommended threshold, further validating the stability of the network centrality measures. Collectively, these findings indicate that, with the exception of bridge-EI in grouped CLPN, the centrality measures in both grouped and non-grouped models are sufficiently stable, with the majority of CS coefficients exceeding the recommended threshold of 0.25. This stability underscores the reliability of the network centrality estimates, supporting their use in subsequent analyses and interpretations. **Figures 9** and **10** illustrate the average correlations between sampled cases and the original sample, with CS coefficients for EI in both grouped and non-grouped CLPN were detailed in **Supplementary Table S9**.

## Discussion

### Main findings

The present study investigated the complex interplay among insomnia, suicidal ideation, and trait depression by employing a cross-lagged panel network (CLPN) approach. Our findings provide several critical insights into these relationships, particularly highlighting the role of trait depression between insomnia on suicidal ideation. The network analysis uncovered intricate temporal dynamics and directional effects among the variables, providing a nuanced understanding of how these factors interact over time.

### Insomnia as a predictor of suicidal ideation

Insomnia emerged as a significant predictor of suicidal ideation, consistent with prior research indicating its role as an independent risk factor for suicide (4, 5, 12, 15, 34, 35). Specifical items, including AIS6 (sense of well-being during the day) and AIS7 (functioning), were identified as critical nodes in the network, exerting strong predictive influences on other variables. This aligns with previous studies showing that sleep disturbances significantly increase the likelihood of suicidal thoughts through various pathways, including emotional dysregulation and cognitive impairments (1, 2, 14, 36).

Our results support the notion that insomnia is not merely a correlate but a potent predictor of suicidal ideation. The robust predictive edges from insomnia symptoms to suicidal ideation factors, such as despair (DSP) and suicide (SUI), underscore the direct influence of sleep disturbances on suicidal thoughts. For instance, AIS7 (functioning) exhibited strong positive weighted correlations with DSP (despair) through AIS6 (sense of well-being during the day), while SUI (suicide) directly predicted DSP (despair), suggesting that specific insomnia features, such as difficulty falling asleep and daytime dysfunction, are strongly associated directly or indirectly with heightened suicidal ideation. The results presented the important role of despair (hopelessness) among the relationships of insomnia and suicidal ideation, consistent with prior studies (37–39) identifying hopelessness as a key mediator linking sleep disturbances to suicide.

Furthermore, the bidirectional relationships observed among certain insomnia symptoms (e.g., AIS4 (total sleep duration) **↔** AIS5 (overall quality of sleep) **↔** SLP (sleep); AIS7 (functioning) **↔** AIS6 (sense of well-being during the day) **↔** DSP (despair)) suggest a feedback loop where poor sleep quality exacerbates emotional distress or irrational cognitive, which in turn worsens sleep problems. This cyclical pattern (40, 41) underscores the importance of addressing insomnia in suicide prevention strategies, since improving sleep quality may disrupt this vicious cycle and mitigate suicide risk.

### The role of trait depression

Trait depression, namely, trait anhedonia (TAN) and trait dysthymia (TDY), played pivotal roles in the non-grouped network structure. TAN (trait anhedonia) was found to be a central node, strongly influenced by multiple variables, including insomnia symptoms and suicidal ideation factors (e.g., despair and optimism). This suggests that TAN (trait anhedonia) may serve as a crucial outcome of insomnia and suicidal thoughts. Despair (DSP) and optimism (OPT), representing hopelessness from opposing perspectives (22), are linked to the core feature of anhedonia (lack of positive emotion) (24), potentially explaining their predictive relationships. Meanwhile, the high in-EI values for TAN (trait anhedonia) also indicate that it is strongly influenced by other variables. For example, the strong directed edges from insomnia symptoms (AIS1 (sleep induction), AIS6 (sense of well-being during the day), AIS7 (functioning)) and suicidal ideation factors (DSP (despair), OPT (optimism)) to TAN (trait anhedonia) highlight how these variables collectively contribute to heightened levels of anhedonia, which in turn increases suicide risk. These do not demonstrate the trait nature of TAN (trait anhedonia). This may be partly attributed to the use of only two waves of data, which limits our capacity to determine whether anhedonia functions as a state-like mediator or a trait-like moderator in leading to suicidal ideation. In other words, insomnia symptoms, despair, and certain depressive traits likely reinforce each other over a longer period, which the current two-wave design cannot fully capture. Future studies employing three or more waves are needed to track how anhedonia evolves over time, which may help to elucidate a more definitive causal pathway to suicidal ideation. However, the connections of anhedonia and several insomnia symptoms agree with previous studies [e.g., daytime insomnia symptoms (42), subjective sleep quality and sleep disturbances (43)], although they mainly refer to state rather than trait. Additionally, insomnia and suicidal ideation may also be chronic (44, 45), with dynamic interactions occurring over short periods (e.g., 1 month). In contrast, TDY (trait dysthymia) exhibited a more peripheral but still influential role, primarily affecting outgoing connections mainly to TAN (trait anhedonia) and OPT (optimism), indicating greater stability than TAN (trait anhedonia), consistent with its invariance (Wilcoxon statistic =35949, *p* =0.669, corrected by false discovery rate (FDR)) across waves. These findings underscore the multifaceted nature of depressive traits in the context of insomnia and suicidal ideation. TDY (trait dysthymia) exhibited a relatively high in-EI value, along with 2 outgoing edges. This suggests TDY (trait dysthymia) is both influenced by and influences other variables, serving as both an outcome and a predictor. Prior studies have reported varying effects of dysthymia on suicide. For example, one study (46) found that dysthymia alone does not increase suicide risk. Another study (47) revealed that dysthymia increases suicide risk when comorbid with personality disorders. Together, these findings suggest TDY (trait dysthymia) may play a dual role, acting as both an outcome of chronic suicidal ideation and a predictor of suicide-related risks.

Comparisons between high and low levels of trait depression in the grouped networks revealed interesting differences. In both TAN (trait anhedonia) and TDY (trait dysthymia) group comparisons, EI value rankings were inconsistent. However, a non-significant correlation was observed in in-EI values between high-TAN and low-TAN groups, with OPT (optimism) playing a key differentiating role. While the influence of OPT (optimism) varied across TDY (trait dysthymia) groups, the overall significant EI correlation suggested it did not substantially alter the network. These results suggest TAN (trait anhedonia) may have a greater impact on the insomnia-suicidal ideation network than TDY (trait dysthymia), through the key role of optimism. This highlights the critical role of diminished positive emotion, rather than distressed emotion, in moderating the insomnia-suicidal ideation relationship. Prior studies support these findings, showing that distressed emotions directly influence suicide (48, 49), while diminished positive emotions indirectly increase suicide risk trough other factors (50). However, this study identifies a novel mechanism, with diminished positive emotions acting as a moderator rather than an indirect predictor. In the high-TAN group, nodes such as AIS2 (awakening during the night), AIS7 (functioning), and OPT (optimism) were more influential in linking insomnia and suicidal ideation, whereas in the low-TAN group, AIS3 (final awakening earlier than desired), AIS8 (sleepiness during the day) and DSP (despair) played a more prominent role. These findings suggest that trait anhedonia levels modulate the bridging roles of specific nodes. Previous studies have identified hopelessness as a key mediator in the insomnia-suicidal ideation relationship (38, 51). However, this study reveals that high and low trait anhedonia differentially influence this mediation, with high levels primarily affecting optimism and low levels primarily affecting hopelessness. This may reflect the greater salience of diminished positive emotions over distressed emotions in high trait anhedonia, consistent with its nature as a reduced drive for reward processing (52). The above points all highlight the need for personalized interventions based on individual trait anhedonia levels. Specifically, interventions targeting diminished optimism may be critical in mitigating the progression from insomnia to suicidal ideation.

### Temporal network approach

The application of the CLPN model in this study sheds light on the understanding of dynamic relationships among insomnia, suicidal ideation, and trait depression over time. By incorporating longitudinal data, the CLPN approach allows for a more comprehensive examination of temporal associations and directional effects (17). This methodological provides a clearer picture of how these variables interact over time, offering valuable insights into potential intervention targets. For instance, the identification of critical nodes, including AIS6 (sense of well-being during the day), AIS7 (functioning), and OPT (optimism), as key predictors and mediators can guide targeted interventions to disrupt harmful feedback loops. The temporal progression of directional effects may suggest causal relationships among the variables.

Stability analyses confirmed the reliability of network centrality measures, supporting their use in further analyses. Robust CS coefficients for in-EI and out-EI across both grouped and non-grouped models underscore the stability of the network structure, enhancing confidence in the findings. However, due to the relatively small sample sizes within each subgroup, the bridge centrality indices remain unstable. This highlights the need for caution when interpreting bridge symptoms at the subgroup level. Moreover, this instability may also suggest the absence of a global mediating factor linking modules across different levels of trait depression.

### Potential cultural and academic factors

The present study focused on Chinese medical undergraduates as the sample, as this population is particularly susceptible to mental health problems such as insomnia and SI due to multiple stress (e.g., academic overload, unclear identity, occupational stress, and irregular sleep patterns) compared to other groups (53, 54). However, compared to global research on this issue, both the cultural context and the intensity of stressors in Chinese settings differ substantially. First, public attitudes toward medical professions are generally harsher than those in Western countries (55, 56). Second, the academic (57) and occupational pressure (58) are significantly greater. As a result, insomnia and SI may be more readily detected in this population. Nevertheless, more cross-cultural analyses need to be conducted. We recommend prospective multi-site collaborations employing harmonized measures across different countries and institutional settings. Additionally, testing measurement invariance for insomnia, suicidal ideation, and depression traits would help to determine whether the model is valid cross-nationally.

### Implications

This study has several key implications. First, they underscore the necessity of addressing insomnia in suicide prevention efforts. Given the strong predictive relationships between insomnia symptoms and suicidal ideation, interventions targeting insomnia may significantly reduce suicide risk. Specifically, “sense of well-being during the day” and “functioning” are two critical symptoms that need early intervention. Given the cognitive nature of these symptoms, cognitive-behavioral therapy for insomnia, which improves sleep quality and reduces depression, is a promising intervention for suicide prevention (2).

Second, the critical role of trait anhedonia underscores the need to assess and treat anhedonia in clinical or public health practice. Interventions that target trait anhedonia, such as behavioral activation (59–61) and mindfulness-based therapies (62, 63), could help disrupt the progression from insomnia symptoms to severe suicidal thoughts. Furthermore, the differential effects of high and low levels of trait depression suggest that personalized treatment plans tailored to individual trait profiles may enhance intervention efficacy.

Finally, the identification of key nodes within the network provides precise symptom-level targets for intervention. For example, optimizing interventions to target nodes with high out-EI values across subgroups, such as AIS6 (sense of well-being during the day) and AIS7 (functioning), could facilitate connectivity between different network nodes, thereby reduce the overall network density and prevent suicide risk.

### Limitations and future directions

Despite the strengths of this study, several limitations should be acknowledged. First, the sample consisted solely of undergraduate students from a medical university, which may limit the generalizability of the findings to broader populations. Although previous studies found similar connection patterns between insomnia and SI in diverse populations, more precise symptom-level directional linkages remain unclear. Therefore, future research should include more diverse samples to validate symptom-level relationships through CLPN approach across different demographic groups.

Second, the use of SIOSS may introduce response bias. On one hand, the reliance on self-reported measures may introduce the possibility of over- or under-estimation of certain symptom facets. On the other hand, although SIOSS have good psychometric properties within Chinese samples, yet its validation in non-Chinese or more diverse populations is still limited. In the future, objective measures of sleep quality, such as actigraphy, could provide more accurate assessments of insomnia symptoms. Additionally, incorporating biological markers of depression and stress could offer a more comprehensive understanding of the underlying mechanisms. As to the issue of cross-cultural validation, the globally used scale for SI, such as Beck Scale for Suicidal Ideation (BSSI), has brought significant ethical issues, especially in nonclinical populations. Future study needs a more generalizable measures in diverse settings and cultural context to ascertain cross-cultural validity.

Third, the cross-lagged panel design, while informative, still does not interpret causality sufficiently. Longitudinal studies with multiple time points, clinical interviews, standardized clinician ratings, and experimental designs are needed to confirm the causal relationships suggested by the current findings. Future research could also explore the moderating effects of other psychological and environmental factors, such as social support and life events, on the relationships between insomnia and suicidal ideation through CLPN analysis.

## Conclusion

In conclusion, this study provides valuable insights into the complex interplay between insomnia, suicidal ideation, and trait depression. By employing a CLPN approach, we have identified critical nodes and pathways that can inform targeted interventions aimed at reducing suicide risk. These findings underscore the importance of addressing sleep disturbances and different roles of depressive traits in suicide prevention efforts, highlighting the need for personalized and comprehensive treatment strategies. Future research should build on these findings by exploring more diverse populations, further elucidating the mechanisms underlying these relationships, and developing effective interventions for individuals at risk of suicide.

## Data Availability

The raw data supporting the conclusions of this article will be made available by the authors, without undue reservation.

## References

[1] McCall WV Black CG. The link between suicide and insomnia: theoretical mechanisms. Curr Psychiatry Rep. (2013) 15:389. doi: 10.1007/s11920-013-0389-9 23949486 PMC3791319

[2] Riemann D Krone LB Wulff K Nissen C. Sleep, insomnia, and depression. Neuropsychopharmacology. (2020) 45:74–89. doi: 10.1038/s41386-019-0411-y 31071719 PMC6879516

[3] DeShong HL Tucker RP. Borderline personality disorder traits and suicide risk: The mediating role of insomnia and nightmares. J Affect Disord. (2019) 244:85–91. doi: 10.1016/j.jad.2018.10.097 30326346

[4] Patel KK Kearns JC Foti D Pigeon WR Kleiman EM Glenn CR. Anhedonia links sleep problems and suicidal thoughts: an intensive longitudinal study in high-risk adolescents. Res Child Adolesc Psychopathol. (2024) 55:331–47. doi: 10.1007/s10802-024-01275-w PMC11913912 39680285

[5] Yiyue Y Kaiqi G Rujie W Honghong L Xu M Yingxue F. Effects of sleep quality on suicide risk in COVID-19 patients: The chain mediating of anxiety and depressive symptoms. Heliyon. (2023) 9:e15051. doi: 10.1016/j.heliyon.2023.e15051 37012905 PMC10060188

[6] Pigeon WR Pinquart M Conner K. Meta-analysis of sleep disturbance and suicidal thoughts and behaviors. J Clin Psychiatry. (2012) 73:e1160–7. doi: 10.4088/JCP.11r07586 23059158

[7] Bernert RA Kim JS Iwata NG Perlis ML. Sleep disturbances as an evidence-based suicide risk factor. Curr Psychiatry Reports. (2015) 17:554. doi: 10.1007/s11920-015-0554-4 PMC6613558 25698339

[8] Anna Karin H Hössjer O Bellocco R Ye W Trolle LY Åkerstedt T. Insomnia in the context of short sleep increases suicide risk. Sleep. (2021) 44:zsaa245. doi: 10.1093/sleep/zsaa245 33216134 PMC8033451

[9] Guo Z Han X Kong T Wu Y Kang Y Liu Y. The mediation effects of nightmares and depression between insomnia and suicidal ideation in young adults. Sci Reports. (2024) 14:9577. doi: 10.1038/s41598-024-58774-5 PMC11052998 38670978

[10] Bae SM Lee YJ Cho IH Kim SJ Im JS Cho SJ. Risk factors for suicidal ideation of the general population. J Korean Med Sci. (2013) 28:602–7. doi: 10.3346/jkms.2013.28.4.602 PMC3617315 23579548

[11] Gong Q Li S Wang S Li H Han L. Sleep and suicidality in school-aged adolescents: A prospective study with 2-year follow-up. Psychiatry Res. (2020) 287:112918. doi: 10.1016/j.psychres.2020.112918 32203752

[12] Alter S Wilson C Sun S Harris RE Wang Z Vitale A. The association of childhood trauma with sleep disturbances and risk of suicide in US veterans. J Psychiatr Res. (2021) 136:54–62. doi: 10.1016/j.jpsychires.2021.01.030 33561736

[13] Darquennes G Wacquier B Loas G Hein M. Suicidal ideations in major depressed subjects: role of the temporal dynamics of anhedonia. Brain Sci. (2023) 13:1065. doi: 10.3390/brainsci13071065 37508997 PMC10377246

[14] Grove JL Smith TW Carlson SE Bryan CJ Crowell SE Czajkowski L. Prospective association between suicide cognitions and emotional responses to a laboratory stressor: The mediating role of nightly subjective sleep quality. J Affect Disord. (2020) 265:77–84. doi: 10.1016/j.jad.2020.01.060 31957695

[15] Geoffroy PA Oquendo MA Courtet P Blanco C Olfson M Peyre H. Sleep complaints are associated with increased suicide risk independently of psychiatric disorders: results from a national 3-year prospective study. Mol Psychiatry. (2021) 26:2126–36. doi: 10.1038/s41380-020-0735-3 32355334

[16] Lu Y Liu Z Luo X Song L Fan T Huang C. The association between insomnia and suicide attempts among Chinese adolescents: a prospective cohort study. BMC Psychol. (2024) 12:777. doi: 10.1186/s40359-024-02273-9 39719641 PMC11669201

[17] Epskamp S Waldorp LJ Mõttus R Borsboom D. The Gaussian Graphical Model in cross-sectional and time-series data. Multivariate Behav Res. (2018) 53:453–80. doi: 10.1080/00273171.2018.1454823 29658809

[18] Epskamp S Borsboom D Fried EI. Estimating psychological networks and their accuracy: A tutorial paper. Behav Res Methods. (2018) 50:195–212. doi: 10.3758/s13428-017-0862-1 28342071 PMC5809547

[19] Funkhouser CJ Chacko AA Correa KA Kaiser AJE Shankman SA. Unique longitudinal relationships between symptoms of psychopathology in youth: A cross-lagged panel network analysis in the ABCD study. J Child Psychol Psychiatry. (2021) 62:184–94. doi: 10.1111/jcpp.13256 PMC7657959 32399985

[20] Soldatos CR Dikeos DG Paparrigopoulos TJ. Athens Insomnia Scale: validation of an instrument based on ICD-10 criteria. J Psychosom Res. (2000) 48:555–60. doi: 10.1016/S0022-3999(00)00095-7 11033374

[21] Xia C Wang D Wu S Ye J. The development of self-rating idea of suicide scale. J Clin Psychiatry. (2002) 12:100–2. doi: 10.3969/j.issn.1005-3220.2002.02.030

[22] Xia C Wang D He X Ye H. Study of self-rating idea undergraduates in the mountain area of southern Zhejiang. Chin J School Health. (2012) 33:144–6.

[23] Xu Z Chen B Li G Dai W. The interference in the suicide ideation of patients with Malignant tumors by mental clinical nursing pathway. Patient Prefer Adherence. (2014) 8:1665–9. doi: 10.2147/PPA.S74132 PMC4262215 25525342

[24] Krohne H Schmukle S Spaderna H Spielberger C. The state-trait depression scales: an international comparison. Anxiety Stress Coping. (2002) 15:105–22. doi: 10.1080/10615800290028422

[25] Lei Z Xu R Deng S Luo Y. Reliability and validity of the Chinese version of State-Trait Depression Scale in college students. Chin Ment Health J. (2011) 25:136–40. doi: 10.3969/j.issn.1000-6729.2011.02.013

[26] IBM-Corp. IBM SPSS Statistics for Windows, Version 25.0. 2017. Armonk, NY: IBM Corp (2017). Available online at: https://www.ibm.com (Accessed October 10, 2023).

[27] R-Core-Team. R: A language and environment for statistical computing. Vienna: R Foundation for Statistical Computing (2024). Available online at: https://cran.r-project.org (Accessed June 15, 2024).

[28] RStudio-Team. RStudio: Integrated development environment for R. Boston, MA: RStudio, PBC (2024). Available online at: http://www.rstudio.com (Accessed June 10, 2024).

[29] Friedman J Hastie T Tibshirani R. Sparse inverse covariance estimation with the graphical lasso. Biostatistics. (2008) 9:432–41. doi: 10.1093/biostatistics/kxm045 PMC3019769 18079126

[30] Friedman JH Hastie T Tibshirani R. Regularization paths for generalized linear models via coordinate descent. J Stat Software. (2010) 33:1–22. doi: 10.18637/jss.v033.i01 PMC2929880 20808728

[31] Epskamp S Cramer AOJ Waldorp LJ Schmittmann VD Borsboom D. qgraph: Network visualizations of relationships in psychometric data. J Stat Software. (2012) 48:1–18. doi: 10.18637/jss.v048.i04

[32] Opsahl T Agneessens F Skvoretz J. Node centrality in weighted networks: Generalizing degree and shortest paths. Soc Netw. (2010) 32:245–51. doi: 10.1016/j.socnet.2010.03.006

[33] Jones PJ Ma R McNally RJ. Bridge centrality: A network approach to understanding comorbidity. Multivariate Behav Res. (2021) 56:353–67. doi: 10.1080/00273171.2019.1614898 31179765

[34] Simmons Z Erickson LD Hedges D Kay DB. Insomnia is associated with frequency of suicidal ideation independent of depression: A replication and extension of findings from the national health and nutrition examination survey. Front Psychiatry. (2020) 11:561564. doi: 10.3389/fpsyt.2020.561564 33192680 PMC7530944

[35] Primack JM Quinn MJ Carskadon MA Holman CS Nazem S Kelsey MR. Longitudinal assessment of the sleep suicide link in Veterans: methods and study protocol. Sleep Adv. (2023) 4:zpad025. doi: 10.1093/sleepadvances/zpad025 37303865 PMC10254730

[36] Killgore WDS Grandner MA Tubbs AS Fernandez FX Doty TJ Capaldi Ii VF. Sleep loss suicidal ideation: the role of trait extraversion. Front Behav Neurosci. (2022) 16:886836. doi: 10.3389/fnbeh.2022.886836 36338878 PMC9630630

[37] Kivelä L Krause-Utz A Mouthaan J Schoorl M de Kleine R Elzinga B. Longitudinal course of suicidal ideation and predictors of its persistence - A NESDA study. J Affect Disord. (2019) 257:365–75. doi: 10.1016/j.jad.2019.07.042 31302526

[38] Kivelä LMM van der Does W Antypa N. Sleep, hopelessness, and suicidal ideation: An ecological momentary assessment and actigraphy study. J Psychiatr Res. (2024) 177:46–52. doi: 10.1016/j.jpsychires.2024.06.039 38972264

[39] Woosley JA Lichstein KL Taylor DJ Riedel BW Bush AJ. Hopelessness mediates the relation between insomnia and suicidal ideation. J Clin Sleep Med. (2014) 10:1223–30. doi: 10.5664/jcsm.4208 PMC4224724 25325598

[40] Liu X Yang Y Liu ZZ Jia CX. Bidirectional associations between sleep problems and suicidal thought/attempt in adolescents: A 3-wave data path analysis. J Affect Disord. (2024) 350:983–90. doi: 10.1016/j.jad.2024.01.153 38244795

[41] Yang J Zhao Y. Examining bidirectional relations between sleep problems and non-suicidal self-injury/suicidal behavior in adolescents: emotion regulation difficulties and externalizing problems as mediators. Eur Child Adolesc Psychiatry. (2024) 33:2397–411. doi: 10.1007/s00787-023-02334-1 38150149

[42] Osorno RA Kaplan K Krystal A Buysse D Edinger J Manber R. Daytime insomnia symptoms negatively predict anhedonia in patients with comorbid major depressive disorder and insomnia disorder. Sleep. (2018) 41:A361–A2. doi: 10.1093/sleep/zsy061.973

[43] Wang X Ren L Zhao X Shi Y Li J Wu W. Network structure of sleep quality and its bridging association with anhedonia in adolescent major depression disorder. Physiol Behav. (2025) 292:114833. doi: 10.1016/j.physbeh.2025.114833 39894190

[44] Duquenne P Samieri C Chambaron S Brindisi MC Kesse-Guyot E Galan P. Chronic insomnia, high trait anxiety and their comorbidity as risk factors for incident type 2 diabetes mellitus. Sci Rep. (2024) 14:11927. doi: 10.1038/s41598-024-62675-y 38789594 PMC11126668

[45] Ronningstam E Schechter M Herbstman B Goldbalatt M. Chronic suicidal ideations: a risk or a protection. Res Psychother. (2024) 27:764. doi: 10.4081/ripppo.2024.764 38904642 PMC11781036

[46] Witte TK Timmons KA Fink E Smith AR Joiner TE. Do major depressive disorder and dysthymic disorder confer differential risk for suicide? J Affect Disord. (2009) 115:69–78. doi: 10.1016/j.jad.2008.09.003 18842304 PMC2674849

[47] Holmstrand C Engström G Träskman-Bendz L. Disentangling dysthymia from major depressive disorder in suicide attempters’ suicidality, comorbidity and symptomatology. Nord J Psychiatry. (2008) 62:25–31. doi: 10.1080/08039480801960164 18389422

[48] Yuan T Fan X Chen W Pan Y Lu Y Lin X. Relationship between adolescent depression and suicidal ideation:the mediating role of insomnia. J Int Psychiatry. (2024) 51:1100–3,15.

[49] Guo S Lu W Wang L Gao Y Wang R Song M. Association between childhood abuse and suicidal ideation in adolescents with depression. Chin J Nervous Ment Dis. (2022) 48:281–5. doi: 10.3969/j.issn.1002-0152.2022.05.005

[50] Carvalho CB Teixeira M Costa R Cordeiro F Cabral JM. The enhancing role of emotion regulation in the links between early positive memories and self-harm and suicidal ideation in adolescence. J Youth Adolesc. (2023) 52:1738–52. doi: 10.1007/s10964-023-01777-8 PMC10275796 37178280

[51] Tucker RP Cramer RJ Langhinrichsen-Rohling J Rodriguez-Cue R Rasmussen S Oakey-Frost N. Insomnia and suicide risk: a multi-study replication and extension among military and high-risk college student samples. Sleep Med. (2021) 85:94–104. doi: 10.1016/j.sleep.2021.06.032 34298228

[52] Xia F Fascianelli V Vishwakarma N Ghinger FG Kwon A Gergues MM. Understanding the neural code of stress to control anhedonia. Nature. (2025) 637:654–62. doi: 10.1038/s41586-024-08241-y PMC11735319 39633053

[53] Xu S Zhao J. Meta-analysis of the mental health status of Chinese medical students. Pract Prevent Med. (2018) 25:463–6,73. doi: 10.3969/j.issn.1006-3110.2018.04.021

[54] Zeng W Chen R Wang X Zhang Q Deng W. Prevalence of mental health problems among medical students in China: A meta-analysis. Med (Baltimore). (2019) 98:e15337. doi: 10.1097/MD.0000000000015337 PMC6504335 31045774

[55] Ma Y Ni X Shi Y Yan C Shi L Li Z. Epidemic characteristics and related risk factors of occupational exposure for pediatric health care workers in Chinese public hospitals: a cross-sectional study. BMC Public Health. (2019) 19:1453. doi: 10.1186/s12889-019-7862-2 31690294 PMC6833173

[56] Sun T Gao L Li F Shi Y Xie F Wang J. Workplace violence, psychological stress, sleep quality and subjective health in Chinese doctors: a large cross-sectional study. BMJ Open. (2017) 7:e017182. doi: 10.1136/bmjopen-2017-017182 PMC5728267 29222134

[57] Li Y Cao L Mo C Tan D Mai T Zhang Z. Prevalence of burnout in medical students in China: A meta-analysis of observational studies. Med (Baltimore). (2021) 100:e26329. doi: 10.1097/MD.0000000000026329 PMC8257868 34190150

[58] Liu Y Lu L Wang WX Liu S Chen HR Gao X. Job Burnout and Occupational Stressors among Chinese Healthcare Professionals at County-Level Health Alliances. Int J Environ Res Public Health. (2020) 17:1848. doi: 10.3390/ijerph17061848 32178394 PMC7142970

[59] Alsayednasser B Widnall E O’Mahen H Wright K Warren F Ladwa A. How well do Cognitive Behavioural Therapy and Behavioural Activation for depression repair anhedonia? A secondary analysis of the COBRA randomized controlled trial. Behav Res Ther. (2022) 159:104185. doi: 10.1016/j.brat.2022.104185 36371903

[60] Cernasov PM Walsh EC Nagy GA Kinard JL Kelley L Phillips RD. A parallel-arm, randomized trial of Behavioral Activation Therapy for anhedonia versus mindfulness-based cognitive therapy for adults with anhedonia. Behav Res Ther. (2024) 182:104620. doi: 10.1016/j.brat.2024.104620 39213738 PMC11519751

[61] Cernasov P Walsh EC Kinard JL Kelley L Phillips R Pisoni A. Multilevel growth curve analyses of behavioral activation for anhedonia (BATA) and mindfulness-based cognitive therapy effects on anhedonia and resting-state functional connectivity: Interim results of a randomized trial. J Affect Disord. (2021) 292:161–71. doi: 10.1016/j.jad.2021.05.054 PMC8282772 34126308

[62] Carlton CN Antezana L Garcia KM Sullivan-Toole H Richey JA. Mindfulness-based stress reduction specifically improves social anhedonia among adults with chronic stress. Affect Sci. (2022) 3:145–59. doi: 10.1007/s42761-021-00085-3 PMC9382999 36046096

[63] Bogaert L van der Gucht K Kuppens P Kock M Schreuder MJ Kuyken W. The effect of universal school-based mindfulness on anhedonia and emotional distress and its underlying mechanisms: A cluster randomised controlled trial via experience sampling in secondary schools. Behav Res Ther. (2023) 169:104405. doi: 10.1016/j.brat.2023.104405 37797436 PMC10938062

