# Supplementary material for: Differential directional effects between insomnia symptoms and suicidal ideation across trait depression levels: a cross-lagged network analysis among Chinese medical undergraduates
Source: Front Psychiatry. 2025 May 21;16:1581827. doi: 10.3389/fpsyt.2025.1581827 (PMC12133867; doi:10.3389/fpsyt.2025.1581827)
Supplement: Supplementary file 1 [file Table1.docx]

Table 1S The edges weights of the network

|  | AIS1 | AIS2 | AIS3 | AIS4 | AIS5 | AIS6 | AIS7 | AIS8 | DSP | OPT | SLP | SUI | TAN | TDY |
| --- | --- | --- | --- | --- | --- | --- | --- | --- | --- | --- | --- | --- | --- | --- |
| AIS1 | 0.000 | 0.088 | 0.018 | 0.125 | 0.041 | 0.012 | 0.026 | 0.038 | 0.011 | 0.000 | 0.188 | -0.036 | 0.348 | 0.000 |
| AIS2 | 0.244 | 0.000 | 0.338 | 0.000 | 0.103 | 0.046 | 0.000 | 0.000 | 0.000 | 0.044 | 0.398 | 0.000 | 0.242 | 0.000 |
| AIS3 | 0.040 | 0.296 | 0.000 | 0.000 | 0.000 | 0.009 | 0.000 | 0.000 | 0.000 | 0.000 | 0.329 | 0.050 | 0.000 | 0.000 |
| AIS4 | 0.137 | 0.000 | 0.000 | 0.000 | 0.324 | 0.000 | 0.105 | 0.068 | 0.173 | 0.000 | 0.000 | -0.026 | 0.084 | 0.000 |
| AIS5 | 0.069 | 0.049 | 0.000 | 0.443 | 0.000 | 0.086 | 0.093 | 0.100 | 0.000 | 0.012 | 0.257 | 0.000 | 0.141 | 0.000 |
| AIS6 | 0.025 | 0.009 | 0.000 | 0.000 | 0.162 | 0.000 | 0.324 | 0.077 | 0.144 | 0.088 | 0.000 | 0.000 | 0.961 | 0.745 |
| AIS7 | 0.101 | 0.000 | 0.000 | 0.220 | 0.163 | 0.302 | 0.000 | 0.373 | 0.000 | 0.003 | 0.166 | 0.000 | 0.331 | 0.657 |
| AIS8 | 0.075 | 0.000 | 0.000 | 0.085 | 0.108 | 0.038 | 0.204 | 0.000 | 0.100 | 0.000 | -0.064 | -0.016 | 0.095 | 0.000 |
| DSP | 0.000 | 0.000 | 0.000 | 0.035 | 0.000 | 0.018 | 0.000 | 0.013 | 0.000 | 0.049 | 0.072 | 0.072 | 0.413 | 0.630 |
| OPT | 0.000 | 0.011 | 0.000 | -0.023 | 0.021 | 0.041 | 0.000 | 0.000 | 0.253 | 0.000 | 0.000 | 0.197 | 1.094 | 0.945 |
| SLP | 0.136 | 0.109 | 0.100 | 0.000 | 0.130 | 0.005 | 0.037 | 0.000 | 0.176 | 0.000 | 0.000 | 0.049 | 0.155 | 0.000 |
| SUI | 0.000 | 0.000 | 0.045 | -0.070 | 0.000 | 0.019 | 0.000 | 0.000 | 0.770 | 0.412 | 0.172 | 0.000 | 0.000 | 0.047 |
| TAN | 0.005 | 0.000 | 0.000 | 0.000 | 0.000 | 0.010 | 0.002 | 0.002 | 0.044 | 0.024 | 0.003 | 0.000 | 0.000 | 0.142 |
| TDY | 0.000 | 0.000 | 0.001 | -0.001 | 0.000 | 0.019 | 0.019 | 0.004 | 0.149 | 0.046 | 0.000 | 0.002 | 0.330 | 0.000 |

Note: AIS, the Athens Insomnia Scale; DSP, the despair factor of Self-rating Idea of Suicide Scale (SIOSS); OPT, the optimism factor of SIOSS; SLP, the sleep factor of SIOSS; SUI, the suicide factor of SIOSS; TAN, the trait anhedonia factor of Trait Depression Scale (TDS); TDY, the trait dysthymia factor of TDS. The row and column names represented the starting and ending points of the directed edges, respectively, meaning, for example, that the value in row X and column Y (representing by “β values” in the main text) represents the standardized predictive coefficient from X at T1 to Y at T2.

Table 2S The raw scores of the in-EI, out-EI, and bridge-EI

| variables | in-EI | out-EI | bridge-EI |
| --- | --- | --- | --- |
| AIS1 | 0.833 | 0.859 | 0.511 |
| AIS2 | 0.562 | 1.413 | 0.683 |
| AIS3 | 0.502 | 0.723 | 0.378 |
| AIS4 | 0.814 | 0.865 | 0.231 |
| AIS5 | 1.052 | 1.251 | 0.410 |
| AIS6 | 0.605 | 2.535 | 1.938 |
| AIS7 | 0.811 | 2.317 | 1.157 |
| AIS8 | 0.675 | 0.624 | 0.114 |
| DSP | 1.820 | 1.302 | 1.109 |
| OPT | 0.678 | 2.538 | 2.088 |
| SLP | 1.521 | 0.897 | 0.673 |
| SUI | 0.291 | 1.395 | 0.041 |
| TAN | 4.194 | 0.232 | 0.090 |
| TDY | 3.165 | 0.570 | 0.239 |

Note: Refer to the notes of Table 1S for the variable names. EI, expected influence.

Table 3S The edges weights of the high-TAN network

|  | AIS1 | AIS2 | AIS3 | AIS4 | AIS5 | AIS6 | AIS7 | AIS8 | DSP | OPT | SLP | SUI |
| --- | --- | --- | --- | --- | --- | --- | --- | --- | --- | --- | --- | --- |
| AIS1 | 0.000 | 0.058 | 0.000 | 0.112 | 0.079 | 0.000 | 0.093 | 0.000 | 0.004 | 0.006 | 0.190 | -0.032 |
| AIS2 | 0.154 | 0.000 | 0.316 | 0.000 | 0.091 | 0.005 | 0.000 | 0.000 | 0.000 | 0.062 | 0.442 | 0.000 |
| AIS3 | 0.000 | 0.271 | 0.000 | 0.000 | 0.000 | 0.045 | 0.000 | 0.000 | 0.000 | 0.000 | 0.317 | 0.033 |
| AIS4 | 0.127 | 0.000 | 0.000 | 0.000 | 0.301 | 0.001 | 0.108 | 0.013 | 0.107 | 0.000 | 0.031 | -0.006 |
| AIS5 | 0.110 | 0.050 | 0.000 | 0.379 | 0.000 | 0.136 | 0.053 | 0.121 | 0.000 | 0.025 | 0.198 | 0.000 |
| AIS6 | 0.000 | 0.000 | 0.002 | 0.000 | 0.203 | 0.000 | 0.332 | 0.035 | 0.339 | 0.225 | 0.000 | 0.000 |
| AIS7 | 0.201 | 0.000 | 0.000 | 0.211 | 0.095 | 0.338 | 0.000 | 0.401 | 0.176 | 0.067 | 0.087 | 0.000 |
| AIS8 | 0.000 | 0.000 | 0.000 | 0.034 | 0.146 | 0.030 | 0.254 | 0.000 | 0.020 | 0.067 | 0.000 | -0.028 |
| DSP | 0.000 | 0.000 | 0.000 | 0.004 | 0.000 | 0.033 | 0.009 | 0.000 | 0.000 | 0.121 | 0.056 | 0.070 |
| OPT | 0.000 | 0.001 | 0.000 | 0.000 | 0.021 | 0.086 | 0.024 | 0.001 | 0.572 | 0.000 | 0.000 | 0.194 |
| SLP | 0.129 | 0.133 | 0.110 | 0.000 | 0.109 | 0.000 | 0.043 | 0.000 | 0.174 | 0.000 | 0.000 | 0.056 |
| SUI | 0.000 | 0.000 | 0.000 | 0.000 | 0.010 | 0.007 | 0.000 | 0.000 | 0.807 | 0.482 | 0.161 | 0.000 |

Note: Refer to the notes of Table 1S.

Table 4S The edges weights of the low-TAN network

|  | AIS1 | AIS2 | AIS3 | AIS4 | AIS5 | AIS6 | AIS7 | AIS8 | DSP | OPT | SLP | SUI |
| --- | --- | --- | --- | --- | --- | --- | --- | --- | --- | --- | --- | --- |
| AIS1 | 0.000 | 0.116 | 0.048 | 0.077 | 0.000 | 0.015 | 0.000 | 0.169 | 0.000 | 0.000 | 0.133 | 0.000 |
| AIS2 | 0.402 | 0.000 | 0.290 | 0.000 | 0.114 | 0.000 | 0.000 | 0.010 | 0.000 | 0.000 | 0.172 | 0.000 |
| AIS3 | 0.144 | 0.270 | 0.000 | 0.000 | 0.000 | 0.000 | -0.113 | 0.096 | 0.199 | 0.016 | 0.208 | 0.025 |
| AIS4 | 0.114 | 0.000 | 0.031 | 0.000 | 0.324 | 0.000 | 0.097 | 0.089 | 0.115 | 0.000 | 0.000 | 0.000 |
| AIS5 | 0.000 | 0.030 | 0.000 | 0.491 | 0.000 | 0.000 | 0.142 | 0.040 | 0.017 | 0.000 | 0.265 | 0.000 |
| AIS6 | 0.115 | 0.004 | -0.088 | 0.000 | 0.000 | 0.000 | 0.392 | 0.158 | 0.459 | 0.000 | 0.361 | 0.000 |
| AIS7 | 0.000 | 0.000 | -0.140 | 0.203 | 0.275 | 0.273 | 0.000 | 0.372 | 0.125 | 0.000 | 0.027 | 0.000 |
| AIS8 | 0.207 | 0.000 | 0.037 | 0.120 | 0.037 | 0.030 | 0.147 | 0.000 | 0.207 | 0.000 | 0.000 | 0.000 |
| DSP | 0.000 | 0.000 | 0.032 | 0.010 | 0.000 | 0.033 | 0.008 | 0.053 | 0.000 | 0.039 | 0.083 | 0.067 |
| OPT | -0.187 | 0.000 | 0.139 | 0.000 | 0.000 | 0.000 | 0.000 | 0.000 | 0.716 | 0.000 | 0.071 | 0.125 |
| SLP | 0.163 | 0.051 | 0.069 | 0.000 | 0.167 | 0.071 | 0.020 | -0.070 | 0.181 | 0.000 | 0.000 | 0.006 |
| SUI | 0.000 | 0.000 | 0.083 | 0.000 | 0.000 | 0.015 | 0.000 | 0.029 | 1.156 | 0.119 | 0.121 | 0.000 |

Note: Refer to the notes of Table 1S.

Table 5S The raw scores of the EI values for high-TAN and low-TAN groups

| variables | high-TAN group | | | low-TAN group | | |
| --- | --- | --- | --- | --- | --- | --- |
|  | in-EI | out-EI | bridge-EI | in-EI | out-EI | bridge-EI |
| AIS1 | 0.722 | 0.511 | 0.169 | 0.958 | 0.559 | 0.133 |
| AIS2 | 0.513 | 1.070 | 0.504 | 0.471 | 0.988 | 0.172 |
| AIS3 | 0.427 | 0.665 | 0.350 | 0.502 | 0.846 | 0.449 |
| AIS4 | 0.740 | 0.682 | 0.131 | 0.901 | 0.771 | 0.115 |
| AIS5 | 1.055 | 1.073 | 0.222 | 0.917 | 0.986 | 0.282 |
| AIS6 | 0.682 | 1.136 | 0.564 | 0.438 | 1.400 | 0.820 |
| AIS7 | 0.917 | 1.575 | 0.329 | 0.693 | 1.137 | 0.153 |
| AIS8 | 0.571 | 0.524 | 0.059 | 0.947 | 0.785 | 0.207 |
| DSP | 2.199 | 0.293 | 0.046 | 3.176 | 0.325 | 0.136 |
| OPT | 1.055 | 0.899 | 0.133 | 0.174 | 0.864 | -0.048 |
| SLP | 1.481 | 0.754 | 0.524 | 1.441 | 0.658 | 0.471 |
| SUI | 0.287 | 1.467 | 0.017 | 0.224 | 1.524 | 0.128 |

Note: Refer to the notes of Table 1S for the variable names. EI, expected influence.

Table 6S The edges weights of the high-TDY network

|  | AIS1 | AIS2 | AIS3 | AIS4 | AIS5 | AIS6 | AIS7 | AIS8 | DSP | OPT | SLP | SUI |
| --- | --- | --- | --- | --- | --- | --- | --- | --- | --- | --- | --- | --- |
| AIS1 | 0.000 | 0.087 | 0.000 | 0.104 | 0.092 | 0.000 | 0.038 | 0.000 | 0.104 | 0.012 | 0.122 | -0.038 |
| AIS2 | 0.218 | 0.000 | 0.307 | 0.000 | 0.103 | 0.043 | 0.000 | 0.000 | -0.142 | 0.002 | 0.477 | 0.000 |
| AIS3 | 0.008 | 0.278 | 0.000 | 0.000 | 0.000 | 0.004 | 0.000 | 0.000 | -0.191 | 0.000 | 0.422 | 0.038 |
| AIS4 | 0.150 | 0.000 | 0.000 | 0.000 | 0.363 | 0.000 | 0.155 | 0.017 | 0.248 | 0.000 | 0.000 | -0.022 |
| AIS5 | 0.136 | 0.041 | 0.000 | 0.376 | 0.000 | 0.100 | 0.038 | 0.128 | 0.000 | 0.000 | 0.166 | 0.000 |
| AIS6 | 0.000 | 0.038 | 0.000 | 0.000 | 0.182 | 0.000 | 0.315 | 0.011 | 0.272 | 0.341 | 0.000 | 0.000 |
| AIS7 | 0.095 | 0.000 | 0.000 | 0.246 | 0.069 | 0.305 | 0.000 | 0.419 | 0.026 | 0.068 | 0.143 | 0.000 |
| AIS8 | 0.000 | 0.000 | 0.000 | 0.036 | 0.191 | 0.003 | 0.302 | 0.000 | 0.045 | 0.055 | 0.000 | -0.019 |
| DSP | 0.000 | 0.000 | 0.000 | 0.006 | 0.000 | 0.009 | 0.000 | 0.000 | 0.000 | 0.099 | 0.061 | 0.075 |
| OPT | 0.000 | 0.000 | 0.000 | 0.000 | 0.007 | 0.099 | 0.030 | 0.000 | 0.494 | 0.000 | 0.000 | 0.187 |
| SLP | 0.077 | 0.130 | 0.124 | 0.000 | 0.097 | 0.000 | 0.062 | 0.000 | 0.256 | 0.000 | 0.000 | 0.058 |
| SUI | 0.000 | 0.000 | 0.000 | -0.014 | 0.007 | 0.000 | 0.000 | 0.000 | 0.779 | 0.400 | 0.134 | 0.000 |

Note: Refer to the notes of Table 1S.

Table 7S The edges weights of the low-TDY network

|  | AIS1 | AIS2 | AIS3 | AIS4 | AIS5 | AIS6 | AIS7 | AIS8 | DSP | OPT | SLP | SUI |
| --- | --- | --- | --- | --- | --- | --- | --- | --- | --- | --- | --- | --- |
| AIS1 | 0.000 | 0.076 | 0.022 | 0.108 | 0.000 | 0.003 | 0.024 | 0.098 | 0.000 | 0.000 | 0.260 | 0.000 |
| AIS2 | 0.245 | 0.000 | 0.310 | 0.000 | 0.090 | 0.000 | 0.000 | 0.075 | 0.000 | 0.000 | 0.234 | 0.000 |
| AIS3 | 0.045 | 0.260 | 0.000 | 0.000 | 0.000 | 0.000 | 0.000 | 0.033 | 0.189 | 0.000 | 0.127 | 0.000 |
| AIS4 | 0.105 | 0.000 | 0.000 | 0.000 | 0.286 | 0.000 | 0.070 | 0.068 | 0.112 | 0.000 | 0.000 | 0.000 |
| AIS5 | 0.000 | 0.024 | 0.000 | 0.497 | 0.000 | 0.029 | 0.136 | 0.035 | -0.151 | 0.000 | 0.337 | 0.000 |
| AIS6 | 0.028 | 0.000 | 0.000 | 0.000 | 0.088 | 0.000 | 0.388 | 0.075 | 0.323 | 0.000 | 0.248 | 0.000 |
| AIS7 | 0.112 | 0.000 | 0.000 | 0.165 | 0.226 | 0.321 | 0.000 | 0.293 | 0.233 | 0.000 | 0.022 | 0.000 |
| AIS8 | 0.153 | 0.026 | 0.016 | 0.084 | 0.042 | 0.028 | 0.124 | 0.000 | 0.134 | 0.000 | -0.089 | 0.000 |
| DSP | 0.000 | 0.000 | 0.017 | 0.022 | -0.024 | 0.024 | 0.028 | 0.010 | 0.000 | 0.000 | 0.095 | 0.008 |
| OPT | -0.076 | 0.005 | 0.000 | 0.000 | 0.000 | 0.000 | 0.000 | 0.000 | 0.666 | 0.000 | 0.103 | 0.000 |
| SLP | 0.218 | 0.067 | 0.045 | 0.000 | 0.175 | 0.062 | 0.004 | 0.000 | 0.201 | 0.000 | 0.000 | 0.000 |
| SUI | -0.008 | 0.061 | 0.045 | 0.000 | 0.000 | 0.082 | 0.000 | 0.000 | 1.244 | 0.000 | 0.151 | 0.000 |

Note: Refer to the notes of Table 1S.

Table 8S The raw scores of the EI values for high-TDY and low-TDY groups

| variables | high-TDY group | | | low-TDY group | | |
| --- | --- | --- | --- | --- | --- | --- |
|  | in-EI | out-EI | bridge-EI | in-EI | out-EI | bridge-EI |
| AIS1 | 0.684 | 0.520 | 0.199 | 0.819 | 0.591 | 0.260 |
| AIS2 | 0.575 | 1.008 | 0.337 | 0.519 | 0.954 | 0.234 |
| AIS3 | 0.430 | 0.559 | 0.269 | 0.454 | 0.654 | 0.315 |
| AIS4 | 0.754 | 0.911 | 0.226 | 0.877 | 0.641 | 0.112 |
| AIS5 | 1.111 | 0.985 | 0.166 | 0.884 | 0.907 | 0.186 |
| AIS6 | 0.563 | 1.159 | 0.612 | 0.549 | 1.149 | 0.570 |
| AIS7 | 0.939 | 1.370 | 0.236 | 0.774 | 1.372 | 0.254 |
| AIS8 | 0.576 | 0.614 | 0.081 | 0.688 | 0.517 | 0.045 |
| DSP | 1.890 | 0.251 | 0.015 | 2.950 | 0.180 | 0.077 |
| OPT | 0.977 | 0.817 | 0.136 | 0.000 | 0.697 | -0.071 |
| SLP | 1.524 | 0.804 | 0.490 | 1.487 | 0.772 | 0.570 |
| SUI | 0.279 | 1.305 | -0.008 | 0.008 | 1.576 | 0.180 |

Note: Refer to the notes of Table 1S for the variable names. EI, expected influence.

Table 9S The raw scores of the EI values for high-TDY and low-TDY groups

| CS | grouped CLPN | non-group CLPN | | | |
| --- | --- | --- | --- | --- | --- |
|  |  | high-TAN | low-TAN | high-TDY | low-TDY |
| in-EI | 0.749 | 0.749 | 0.671 | 0.674 | 0.751 |
| out-EI | 0.749 | 0.595 | 0.361 | 0.362 | 0.516 |
| bridge-EI | 0.516 | 0.206 | 0.129 | 0.204 | 0.206 |

Note: CS, correlation stability; CLPN, cross-lagged panel network; TAN, the trait anhedonia factor of TDS; TDY, the trait dysthymia factor of TDS; EI, expected influence.


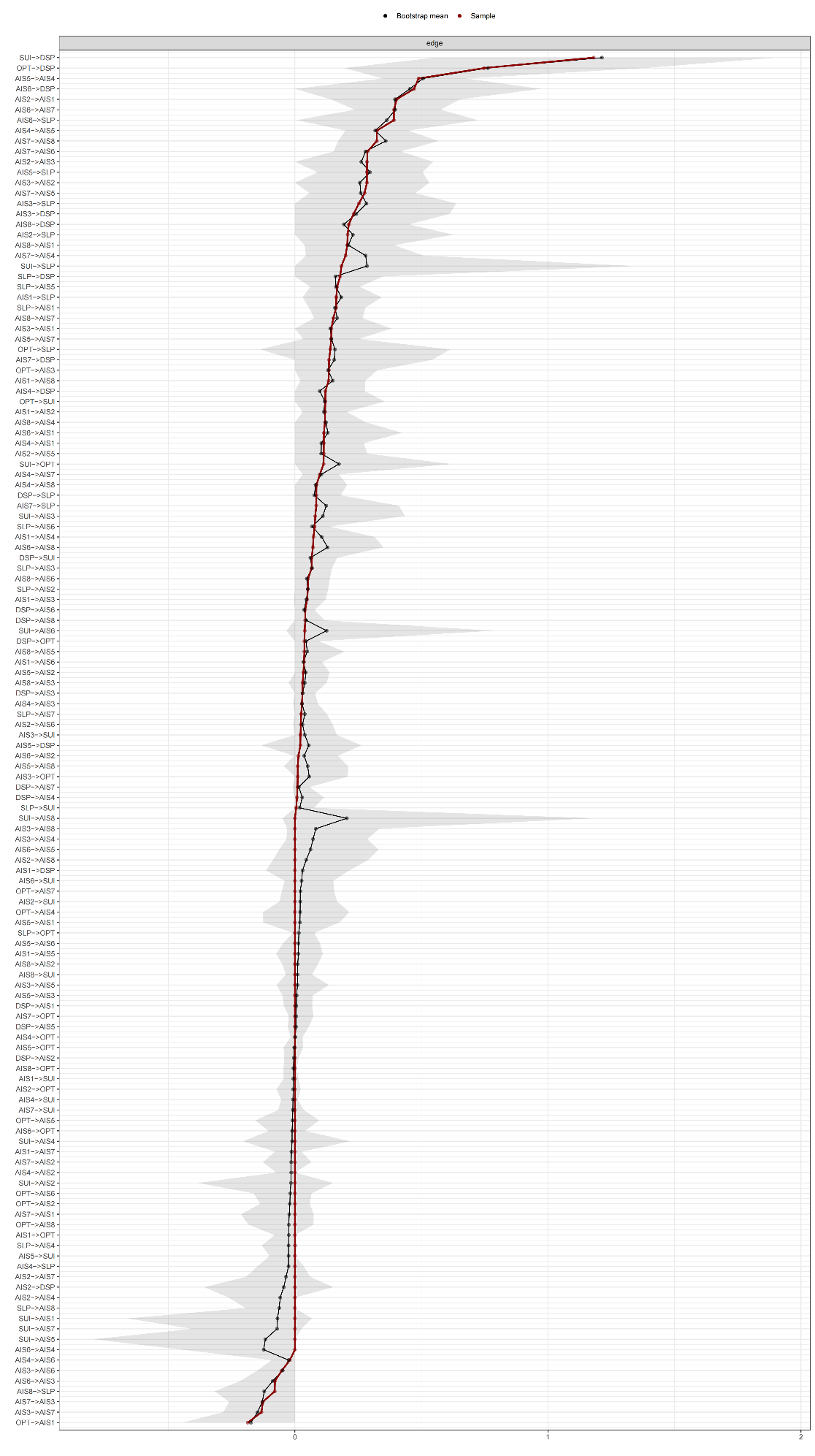


Figure 1S Bootstrap means of the edge weights for low-TAN group.

Note: Refer to the notes of Table 1S for the variable names.


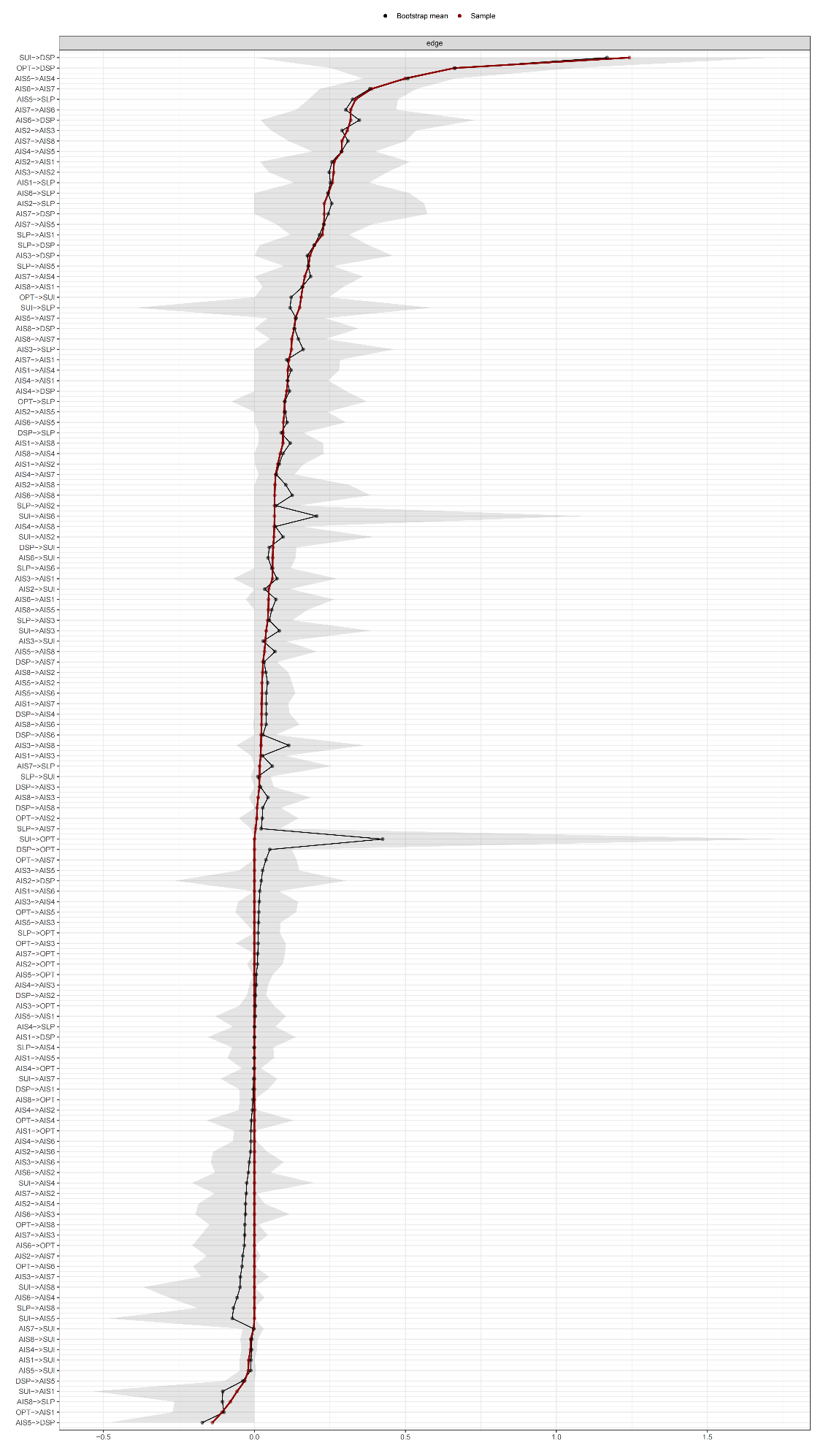


Figure 2S Bootstrap means of the edge weights for low-TDY group

Note: Refer to the notes of Table 1S for the variable names.
